# Long Non-coding RNA SNHG12 Functions as a Competing Endogenous RNA to Regulate MDM4 Expression by Sponging miR-129-5p in Clear Cell Renal Cell Carcinoma

**DOI:** 10.3389/fonc.2019.01260

**Published:** 2019-11-22

**Authors:** Zhipeng Wu, Dongming Chen, Kai Wang, Changchun Cao, Xianlin Xu

**Affiliations:** Department of Urology, The Affiliated Sir Run Run Hospital of Nanjing Medical University, Nanjing, China

**Keywords:** clear cell renal cell carcinoma, SNHG12, miR-129-5p, MDM4, ceRNA

## Abstract

Clear cell renal cell carcinoma (ccRCC), the most common histological subtype of kidney cancer, shows poor prognosis, and non-sensitivity to radiotherapy or chemotherapy. The lncRNA small nucleolar RNA host gene 12 (SNHG12) has been revealed to play a carcinogenic role in various neoplasms, but the underlying mechanism in ccRCC is still unclear. To explore the potential role of SNHG12 in ccRCC, the data downloaded from the Cancer Genome Atlas (TCGA) and International Cancer Genome Consortium (ICGC) Data Portal was used to compare the expression of SNHG12 in tumors and adjacent normal tissues. MRNA microarray and quantitative real-time PCR revealed that SNHG12 was overexpressed in the ccRCC tissues and cell lines. Functional inhibition of SNHG12 suppressed the viability and mobility of ccRCC cells. Mechanistically, dual luciferase assay and RNA immunoprecipitation (RIP) assay showed that miR-129-5p could bind to SNHG12 directly. There was a negative relationship between SNHG12 and miR-129-5p. What's more, we used bioinformatics-based prediction software to predict the target genes of miR-129-5p. Through data analysis and experimental verification, we found MDM4, a regulatory factor in p53 pathway, was involved in this ceRNA network. Our findings demonstrated that SNHG12 served as a sponge for miR-129-5p to regulate the expression of MDM4 and p53 pathway in the development of ccRCC.

## Introduction

Renal cell carcinoma (RCC) arises from renal tubular epithelial cells, accounting for more than 90% of all renal malignancies (1, 2). Most of RCCs occur as clear cell renal cell carcinoma (ccRCC) (75–80%), then papillary RCC (10–15%) and chromophobe RCC (5–10%) (2). Surgical resection is the main treatment option for local ccRCC, but still challenged by a relapse rate up to 20% (3, 4). Without sensitivity to radiotherapy or chemotherapy, and effective early diagnostic methods, about 20–30% of ccRCC patients demonstrate distant metastasis at the initial diagnosis (5). Therefore, it is urgent to find a new effective diagnostic tool or therapeutic target of ccRCC.

lncRNAs are transcripts more than 200 nucleotides in length, and most of them do not have the ability to encode proteins (6, 7). Only 3% of the human genome are RNA-encoding proteins. LncRNAs, once considered as the functionless by-products of transcription, have been found irreplaceable in the occurrence and development of various diseases. Tumor epigenetics, signaling pathway regulation and their interactive regulation as guiding, decoy and signaling molecules are all associated with the abnormal expression of lncRNAs. Besides, cellular behaviors of tumor cells and different stages of tumor progression are closely correlated with abnormally expressed lncRNAs. For example, H19 promotes mucosal regeneration in inflamed intestinal tissues (8); CCR5AS regulates the outcome of HIV disease (9). LncRNAs also show outstanding effects in tumorigenesis (10–12). PXN-AS1-L can degrade mRNA by sponging related miRNA (13). HOTAIR may lead to epigenetic modification by binding to the methyltransferase (14).

SNHG12 located in 1p35.3 has been proven exert oncogenic (15–17), but its role in ccRCC is still unclear. In this paper, we validated the relationship between SNHG12 and ccRCC by using bioinformatics prediction model and experimental data. We found SNHG12 could inhibit the carcinogenicity of ccRCC cells by targeting miR-129-5p and regulate P53 pathway through MDM4.

## Materials and Methods

### Data Collection and Analysis

Genome-wide expression profile of ccRCC patients was downloaded from the UCSC Xena platform (bioRxiv 326470; https://doi.org/10.1101/326470) on “atacseq.xenahubs.net” by choosing the option of “GDC TCGA Kidney Clear Cell Carcinoma (KIRC)”. Related data for SNHG12, miR-129-5p and MDM4 in 71 normal controls and 508 ccRCC tissues were all obtained from this website.

A total of 136 Sequence-based Gene Expression arrays of 91 renal cell carcinoma patients were downloaded from ICGC Data Portal (18) in RENAL CELL CANCER - EU/FR part (https://dcc.icgc.org/projects/RECA-EU).

LncBase Predicted v.2 (19) was used to screen out potential miRNAs binding to SNHG12. In the screening, we set the threshold to 0.9 and the tissue type to kidney, and 15 candidate miRNAs were screened out.

StarBase v2.0 (20), a bioinformatic tool based on various prediction database, was used to explore mRNAs interaction with miR-129-5p. We screened out 1,350 genes which may target miR-129-5p.

### Cell Culture

RCC cell lines (786-O, CAKI-1, ACHN, and 769-P) and renal tubular epithelial cell line (HK2) were purchased from the American Type Culture Collection (ATCC, Manassas VA, USA). The cells were cultured in the medium (HK2 cells in DMEM/F12, 786-O and 769-P cells in RPMI 1640, CAKI-1 cells in McCoy's 5A and ACHN cells in DMEM) containing 10% FBS and 1% penicillin-streptomycin at 37°C in a humidified air with 5% carbon dioxide.

### Cell Transfection

Small interfering RNAs targeting SNHG12 (si-SNHG12), si-NC (negative control), miR-129-5p mimics, and inhibitors were purchased from GenePharma (Shanghai, China). All of them were transfected using Lipofectamine 3000 (Invitrogen, Carlsbad, CA, USA).

### RNA Extraction and Quantitative Real-Time PCR (qRT-PCR) Detection

RNA was separated from collected cells using TRIzol. RNA concentration was measured using NanoDrop 2000 Spectrophotometer (Thermo Scientific, Wilmington, DE, USA). Reverse Transcription Kit (Takara, Tokyo, Japan) was used for reverse transcription of extracted RNA. QRT-PCR was then performed with SYBR Premix Ex Taq TM (Vazyme, China) on Light Cycler 480 (Roche, Switzerland). The expression levels of mRNA were quantified by the comparative cycle threshold (CT). The CT values of mRNA and miRNA were then used to analyze the fold changes of RNA by the 2^−ΔΔ^Ct methods (mRNA and miRNA were normalized by GAPDH and U6, respectively). All the PCR primers were listed in Table 1.

**Table 1:** Primers for real-time PCR.

**Genes**	**Forward (5^**′**^-3^**′**^)**	**Reverse (5^**′**^-3^**′**^)**
SNHG12	TCTGGTGATCGAGGACTTCC	ACCTCCTCAGTATCACACACT
MiR-129-5p	ACACTCCAGCTGGGCTTTTTGCGGTCTGG	CTCAACTGGTGTCGTGGAGTCGGCAATTCAGTTGAGGCAAGCCC
MDM4	TCTCGCTCTCGCACAGGATCACA	AACCACCAAGGCAGGCCAGCTA
GAPDH	GCACCGTCAAGGCTGAGAAC	GGATCTCGCTCCTGGAAGATG
U6	CTCGCTTCGGCAGCACA	AACGCTTCACGAATTTGCGT

### Cell Proliferation Assay

CCK8 (Dojindo, Japan) assay was performed. The cells were cultivated on a 96-well plate (5.0 × 103 cells per well) for 24 h after transfection. TECAN infinite M200 Multimode microplate reader (Tecan, Mechelen, Belgium) was then employed after an hour of incubation in CCK8 at the absorbance of 450 nm.

EdU proliferation assay (RiboBio, Nanjing, China) was conducted to detect the proliferation of the transfected cell. The cells were fixed by 4% paraformaldehyde for half an hour after being treated with 50 μM EdU for 2 h. Following Apollo staining and DAPI staining, a fluorescent microscope was adopted to observe the EdU positive cells.

### Scratch Wound Assay

This essay aimed to assess the migration abilities of cells after transfection. The cells grown in 6-well plates at a density of 70% were transfected with siRNA. When the cells were dense in the field of microscopic view (usually 24 h after the transfection), three standardized wound scratches per well were made by a sterile 10 μL pipette tip. The cells were cultured with serum-free medium after scratching. A phase-contrast microscope was adopted to photograph the size of the wound at different time frames (0 and 24 h).

### Cell Invasion Assay

Matrigel (200 mg/ml) was added into transwell chambers (Corning, NY, USA) 12 h before the experiment. The cell density was adjusted to 1.0 × 10^4^ cells per chamber. The cells were cultured in a serum-free medium in the upper chambers for 24 h, and 600 μL of medium containing 10% FBS was put in the lower chambers. The cells on the upper surface were removed by cotton swabs. Those having invaded through the membranes were fixed with 4% paraformaldehyde for 20 min and stained with 0.1% crystal violet for 30 min. A phase-contrast microscope at 20× magnification was employed to count the number of stained cells (5 different views per well).

### Flow Cytometry Analysis

The cells seeded in 6-well-plates for 60–70% confluence were firstly synchronized at the G1/S boundary by serum-free medium for 24 h before transfection. After being transfected for 24 h, the cells were suspended by trypsin without EDTA and washed by phosphate-buffered saline (PBS) twice. For the cell apoptosis, 5 μl of FITC Annexin V and 5 μl of propidium iodide (BD Biopharmingen, NJ, USA) were immediately added to the transfected cells suspending in 300 μl of binding buffer for 15 min in dark. In the cell cycle analysis, the transfected cells were allowed to stay in the 70% ethanol at 4°C for more than 24 h firstly, stained in dark for 15 min in 500 μl propidium oxide staining solution, and then detected by a flow cytometer (FACScan; BD Biosciences, USA) using Cell Quest software (BD Biosciences).

### Dual-Luciferase Assay

The cells were cultured in a 24-well plate until showing 60–70% confluence. Plasmids (MUT type or WT type) and miRNAs (miR-129-5p mimic or negative control) were then co-transfected into the cells. The cells were collected after 48 h for luciferase detection with the dual-luciferase reporter gene assay system (Promega).

### RNA Immunoprecipitation (RIP) Assay

RIP assay was performed using Magna RIP-Kit (Millipore, Bedford, MA, USA). The RNAs used for qRT-PCR analysis were extracted by human anti-Ago2 antibodies (Abcam, ab32381, Shanghai, China) or negative control IgG (Millipore, Billerica, MA, USA), respectively.

### Western Blot Assay

Proteins were isolated from transfected cells by RIPA lysis buffer (Beyotime, Nantong, China) containing 0.5% PMSF. The total proteins (50 μg per protein) were separated on 10% sodium dodecyl sulfate-polyacrylamide gel electrophoresis (SDS-PAGE) and transferred to PVDF membranes (Millipore, Billerica, MA, USA). The membranes were incubated for 1 h in blocking solution (Beyotime) at room temperature and then immunoblotted overnight at 4°C with the following primary antibodies: anti-MDMX (1:1,000, Proteintech Group, Rosemont, IL, USA), anti-P53 (1:1,000, Proteintech Group, Rosemont, IL, USA), anti-P21 (1:1,000, Proteintech Group, Rosemont, IL, USA) and anti-GAPDH (1:1,000, Beyotime). And enhanced chemiluminescence reagent kit (Millipore, Billerica, MA, USA) was utilized for exposure after the blot incubated with secondary antibody (Beyotime) for 1 h.

### Statistical Processing

SPSS 22.0 (SPSS Inc. Chicago, IL, USA) and Graphpad Prism 7 (GraphPad Software Inc., CA, USA) was used for data analysis. Experiments were carried out in triplicate and the data was displayed as mean ± SD. Univariate and multivariate Cox regression analyses were used to analyze correlations between variables and survival. Correlations between SNHG12 expression and clinicopathological variables of ccRCC patients were studied by Chi-square test. Pearson's correlation analysis was used to analyze the mutual correlations between SNHG12, miR-129-5p, and MDM4. Comparisons between groups were conducted using Student's *t*-test or one-way ANOVA. Statistical significance was considered when *p* < 0.05.

## Results

### SNHG12 Was Overexpressed in ccRCC

**Figure 1:**
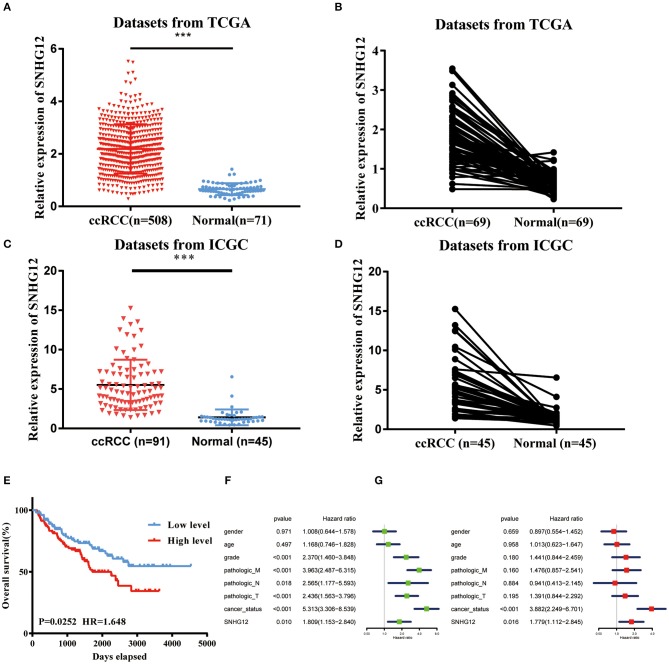
SNHG12 was upregulated in ccRCC tissues. **(A)** Relative expression of SNHG12 in ccRCC tissues compared with normal tissues was analyzed using TCGA data. **(B)** Relative expression of SNHG12 in 69 pairs matched ccRCC tissues and normal tissues in TCGA data. **(C)** Relative expression of SNHG12 in ccRCC tissues compared with normal tissues was analyzed using IGCA data. **(D)** Relative expression of SNHG12 in 45 pairs matched ccRCC tissues and normal tissues in TCGA data. **(E)** Kaplan-Meier survival curves according to SNHG12 expression levels in TCGA data.Univariate **(F)** and multivariate **(G)** Cox regression analysis of clinical parameters and SNHG12 in TCGA database of ccRCC patients. Clinical variables included gender (Female vs. Male), age (<60 vs. > = 60), grade (G1+G2 vs. G3+G4), pathologic_M (M0 vs. M1), pathologic_N (N0 vs. N1), pathologic_T (T1+T2 vs. T3+T4), cancer_status (Tumor free vs. With tumor) and SNHG12 (Low level vs. High level). Error bars stand for the mean ± SD of at least triplicate experiments. ^***^P < 0.001.

To explore the role of SNHG12 in ccRCC, we firstly downloaded the RNA sequencing (RNA-seq) dataset of 508 ccRCC tissues and 71 normal tissues from the UCSC Xena platform in TCGA. The expression level of SNHG12 was significantly higher in ccRCC tissues than in normal tissues (Figure 1A). Besides, this level was upregulated in tumor tissues compared to that of adjacent normal tissues in 69 patients (Figure 1B). Meanwhile, we introduced the expression of SNHG12 of ccRCC patients in the ICGC database and obtained the same results (Figures 1C,D). The clinical data of 207 patients from TCGA (301 patients were excluded because of inadequate clinical information) were divided into two subgroups based on the median expression level of SNHG12. It seemed that patients with different levels of SNHG12 showed different clinical outcomes (Figure 1E). Furthermore, univariate and multivariate Cox regression analyses revealed that SNHG12 was an independent biomarker for ccRCC patients (Figures 1F,G). Besides, Chi-square test results suggested that there was a correlation between lymph node metastasis and the expression level of SNHG12 (Table 2, *p* <0.05). These findings illustrated that SNHG12 might function in ccRCC.

**Table 2:** Clinical characteristics of study population from TCGA.

**Variable**	**Total no. (*n* = 207)**	**Relative SNHG12 expression**		***P* value**
		**Low (*n* = 103)**	**High (*n* = 104)**	
**Correlation between SNHG12 expression and clinicopathological variables**				
**of ccRCC patients**				
**Gender**				*P* = 0.362
Female	80	43	37	
Male	127	60	67	
**Diagnosis age (years)**				*P* = 0.234
<60	95	43	52	
≥60	112	60	52	
**Grade**				*P* = 0.840
G1+G2	91	46	45	
G3+G4	116	57	59	
**Pathologic M**				*P* = 0.285
M0	171	88	83	
M1	36	15	21	
**Pathologic N**				***P*** **=** **0.031**
N0	196	101	95	
N1	11	2	9	
**Pathologic T**				*P* = 0.714
T1+T2	122	62	60	
T3+T4	85	41	44	
**Person neoplasm cancer status**				*P* = 0.499
Tumor free	134	69	65	
With tumor	73	34	39	

### Downregulation of SNHG12 Inhibited the Viability and Mobility of ccRCC *in vitro*

**Figure 2:**
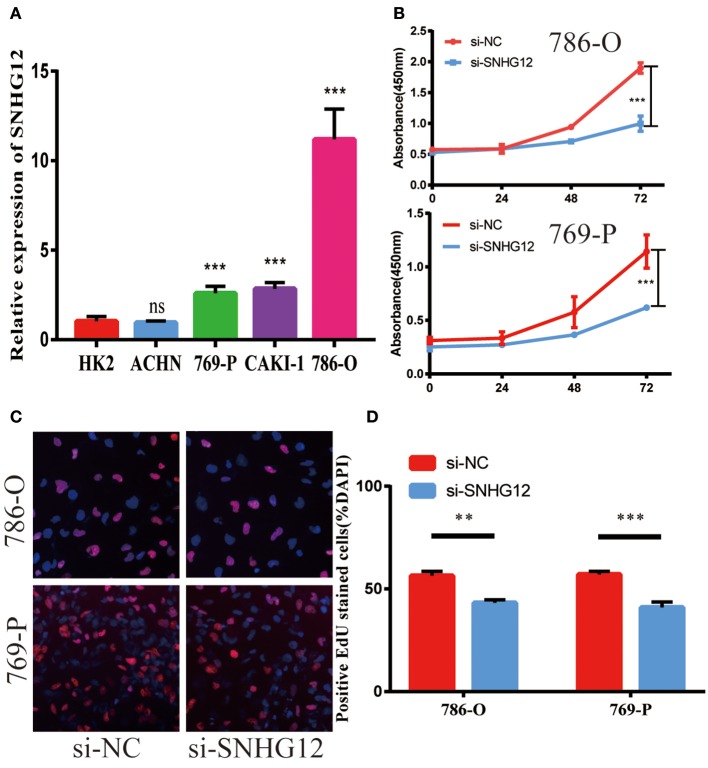
Effects of SNHG12 on ccRCC cells' proliferation ability. **(A)** Relative expression of SNHG12 in renal tubular epithelial cells (HK2) and ccRCC cell lines. **(B)** CCK8 assays were performed to determine the proliferation ability of si-SNHG12-transfected cells. **(C,D)** Edu staining assays were performed to determine the proliferation ability of si-SNHG12-transfected cells. Error bars stand for the mean ± SD of at least triplicate experiments. ^**^P < 0.01; ^***^P < 0.001.

**Figure 3:**
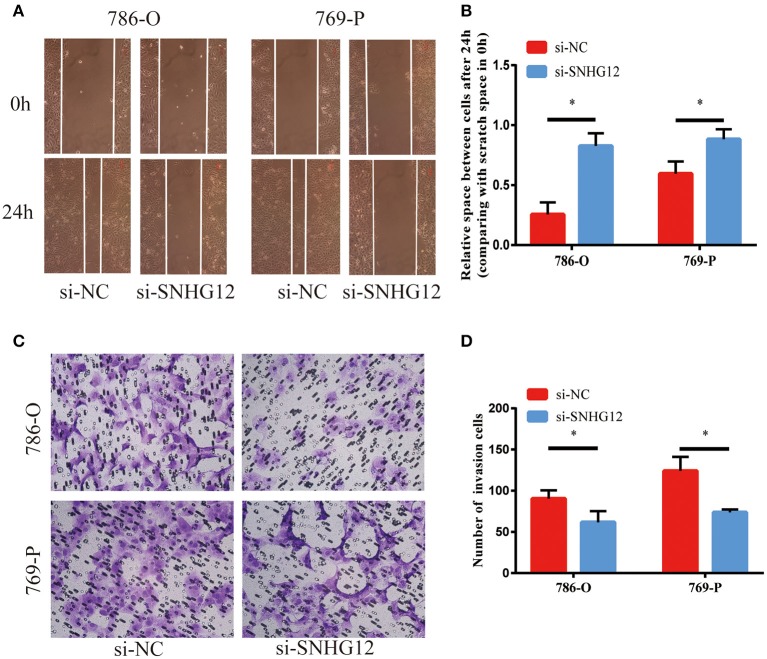
Effects of SNHG12 on ccRCC cells'mobility *in vitro*. **(A,B)** Scratch wound assays were conducted to detect the migration abilities of si-SNHG12-transfected cells. Scratch wound assays were photographed at a magnification of 10X. **(C,D)** Transwell assays were conducted to detect the invasion abilities of si-SNHG12-transfected cells. Transwell assays were photographed at a magnification of 20X. Error bars stand for the mean ± SD of at least triplicate experiments. ^*^P < 0.05.

**Figure 4:**
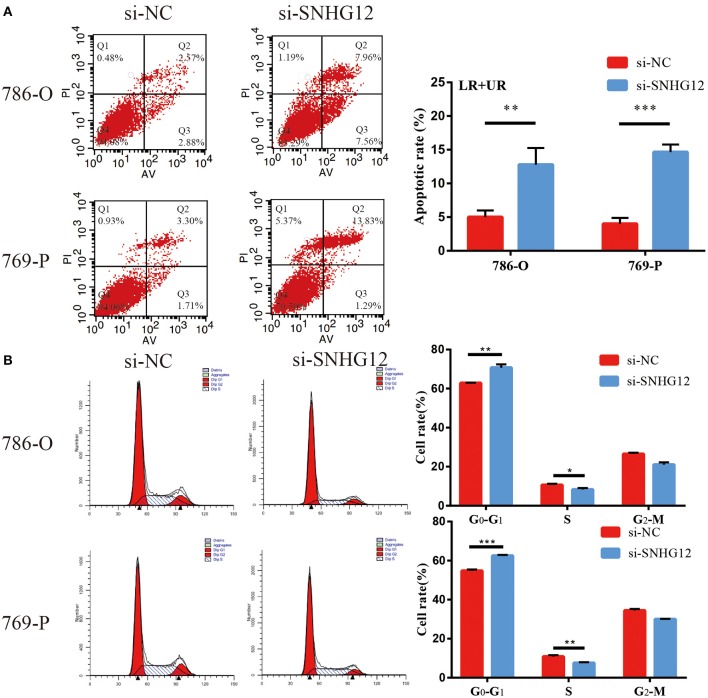
Effects of SNHG12 on ccRCC cells apoptosis and cell cycle. **(A)** Flow cytometry was used to detect the apoptotic rates (LR + UR) of cells. LR, early apoptotic cells; UR, terminal apoptotic cells. **(B)** Flow cytometry assay was employed to analyze the cell cycle. Error bars stand for the mean ± SD of at least triplicate experiments. ^*^P < 0.05; ^**^P < 0.01; ^***^P < 0.001.

To delve deeper into the function of SNHG12 in ccRCC, we detected the expression of SNHG12 in ccRCC cell lines (ACHN, 769-P, Caki-1, 786-O) and renal tubular epithelial cells (HK2) using qRT-PCR. Compared with HK2, the expression of SNHG12 was increased in 786-O, Caki-1 and 769-P, but did not change in ACHN (Figure 2A). 786-O and 769-P were then subjected to experiments *in vitro*. We reduced the level of SNHG12 in the cells by transfecting it with siRNA. CCK-8 and EdU assays were performed firstly after transfection. For one thing, the growth curve showed that the downregulation of SNHG12 visibly suppressed cell proliferation (Figure 2B). For another, EdU positive cells were decreased in si-SNHG12 group, compared with NC group (Figures 2C,D). And then, scratch wound assay detected that cell migration was decreased after being transfected si-SNHG12 (Figures 3A,B). Meanwhile, transwell assays suggested that cell invasion was largely reduced in the si-SNHG12 group (Figures 3C,D). Flow cytometric assay found that the cells in the group of si-SNHG12 exhibited higher apoptosis rate than those in the si-NC group (Figure 4A). More cells were arrested in the G1 phase and fewer cells in the G2 and S phases compared with those transfected with negative control (Figure 4B). Collectively, knockdown of SNHG12 inhibited the viability and mobility of ccRCC *in vitro*.

### SNHG12 Served As a Sponge for miR-129-5p in ccRCC

**Figure 5:**
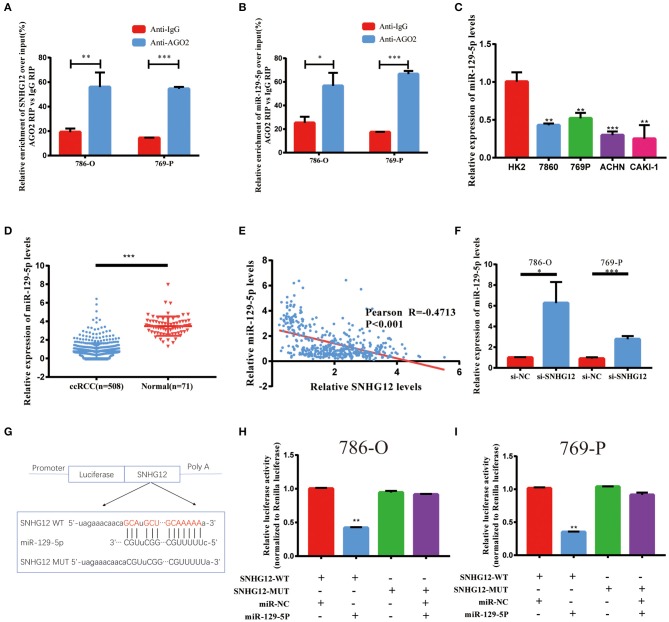
Regulation relationship between SNHG12 and miR-129-5p. **(A)** RNA immunoprecipitation (RIP) experiments for SNHG12 in 786-O and 769-P cell lines. **(B)** RIP experiments for miR-129-5p in 786-O and 769-P cell lines. **(C)** QRT-PCR analysis of miR-129-5p expression in different cell lines. **(D)** Relative expression of miR-129-5p in ccRCC tissues compared with normal tissues was analyzed using TCGA data. **(E)** Pearson correlation analysis between SNHG12 and miR-129-5p expression levels using data of ccRCC patients from TCGA. **(F)** QRT-PCR analysis of miR-129-5p expression in 786-O and 769-P cell linestransfected with si-NC and si-SNHG12. **(G)** Predicted binding site of miR-129-5p in SNHG12 using LncBase Predicted v.2. **(H,I)** Luciferase reporter plasmid containing wild-type (WT)786-O and 769-P cell lines or mutant (Mut) SNHG12 were co-transfected into cells with miR-129-5p in parallel with an empty plasmid vector.Values represent the mean ± SD of three independent experiments.^*^P < 0.05; ^**^P < 0.01; ^***^P < 0.001.

It has been reported that SNHG12 was mainly located in the cytoplasm (21). Thus, we hypothesized that SNHG12 can form an RNA-induced silencing complex (RISC) with miRNAs in ccRCC. RIP assay was performed utilizing the antibody against Ago2. The RIP assay revealed that SNHG12 was significantly enriched in ccRCC cells (Figure 5A). DIANA-LncBase v2 screened out 15 candidate miRNAs binding to SNHG12 with a high predict score (>0.9). Among them, miR-129-5p was found significantly enriched in ccRCC cells by RIP assay (Figure 5B). Besides, miR-129-5p showed a low expression level in ccRCC cell lines compared with that of HK2 (Figure 5C). The expression of miR-129-5p also showed a decreasing trend in tumor tissues in TCGA (Figure 5D). Pearson correlation analysis presented a negative correlation between SNHG12 and miR-129-5p (Figure 5E). What's more, the cells transfected with si-SNHG12 showed an increase expression of miR-129-5p (Figure 5F). Finally, we demonstrated the bond between SNHG12 and miR-129-5p by luciferase reporter gene experiments (Figures 5G–I). Together, SNHG12 might function through regulating the expression of miR-129-5p, with. SNHG12 serving as a sponge.

### MDM4 Was Targeted by miR-129-5p and Regulated by SNHG12

**Figure 6:**
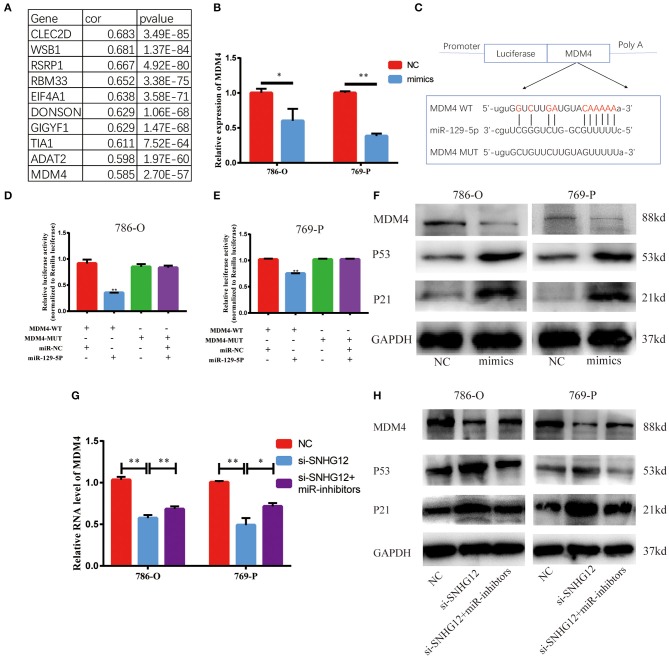
MDM4 is a target gene of miR-129-5p and is restrained by si-SNHG12. **(A)** Pearson correlation analysis between SNHG12 and top 10 candidate genes predicted by StarBase v2.0. **(B)** QRT-PCR result of MDM4 in cells transfected with NC or miR-129-5p mimics. **(C)** Schematic view of miR-129-5p putative targeting site in the WT and Mut 3' UTR of MDM4. **(D,E)** Luciferase activity assay in cells transfected with luciferase report plasmids containing MDM4 3' UTR (WT or Mut), and control miRNA or miR-129-5p. **(F)** The western blot of MDM4 in cells transfected with NC or miR-129-5p mimics. **(G)** and **(H)** MDM4 mRNA and protein level in cells after transfecting NC, si-SNHG12 individually or co-transfected with miR-129-5p inhibitors and si-SNHG12. Values represent the mean ± SD of three independent experiments. ^*^P < 0.05; ^**^P < 0.01.

StarBase v2.0 was used to predict possible target genes in SNHG12-miR-129-5p axis. This bioinformatic tool showed 1,350 potential target genes of miR-129-5p. In ceRNA network, there is usually a positive correlation between lncRNAs and target genes. Thus, we calculated the Pearson correlation efficiency between SNHG12 and 1350 potential target genes base on TCGA datasets, and top 10 genes were selected as candidate genes (Figure 6A). According to the results of qRT-PCR, the expression level of MDM4 decreased when the cells were transfected with the mimics of miR-129-5p (Figure 6B). The dual-luciferase reporter gene assay was conducted to verify the bond between miR-129-5p and MDM4 (Figure 6C). Luciferase activity was significantly abated in the cells co-transfected with MDM4-WT and miR-129-5p mimics (Figures 6D,E). Besides, we measured MDM4 protein levels when miR-129-5p was overexpressed. The level of miR-129-5p increased, the protein level of MDM4 decreased, and p53 and p21 expression were up-regulated (Figure 6F, Figure S1). Furthermore, si-SNHG12 and miR-129-5p were co-transfected, and the other two groups were transfected with NC and si-SNHG12, respectively. The mRNA level of MDM4 in three groups were measured by qRT-PCR (Figure 6G). The results of western blot revealed that the protein level of MDM4 was down-regulated in the si-SNHG12 group and up-regulated in the co-transfection group, with p53 and p21 showing the reverse trend (Figure 6H, Figure S2). We confirmed that MDM4 was involved in the SNHG12-miR-129-5p regulatory axis. In short, SNHG12 regulated the expression of MDM4 through miR-129-5p.

## Discussion

Increasing evidence showed that lncRNAs exert crucial influence on the development of carcinomas. It is reported that SNHG12 accelerates the tumorigenesis of prostate cancer via sponging miR-133b (15) and promotes the progression of the cervical cancer via modulating miR-125b/STAT3 axis (22). In our research, SNHG12 was upregulated in ccRCC. Silencing SNHG12 inhibited the proliferation, migration and invasion of ccRCC cells.

MDM4 encodes a nuclear protein that inhibits p53 by binding to its transcriptional activation domain at the N-terminus. Therefore, MDM4 can suppress the transactivation and apoptosis-causing function of p53 (23). It has been reported that MDM4 is highly expressed in various kinds of cancers and is regulated by miRNAs (24–26). Therefore, MDM4 may act as an anticancer target (27, 28). In our research, MDM4 expression ascended in ccRCC and sharply decreased when transfected with si-SNHG12 or miR-129-5p mimics, followed by the up-regulated protein levels of p53 and its downstream gene p21. It has been reported that p53 and p21 could lead to cell cycle arrest in the G1 phase (29), which is also confirmed by our analysis. Thus, we concluded that p53 might initiate the inhibitory effect of si-SNHG12 on cell proliferation. Taken together, SNHG12 exerts its functions via regulating p53 signaling pathway through SNHG12/miR-129-5p/MDM4 axis.

Shortages are also seen in our research. Firstly, because of the limited number of ccRCC samples, we cannot confirm the universality our results that are only confined in TCGA datasets and ICGC Data Portal. Besides, we should remain cautious about the relationship between SNHG12 and patient survival before there is sufficient evidence. Secondly, our experiment can explain the effect of SNHG12 on cycle arrest and apoptosis, but not on the migration and invasion of ccRCC cells. Finally, it would be better if we could verify our findings in animal experiments.

In conclusion, SNHG12 is overexpressed in ccRCC and acts on the development of ccRCC via regulating p53 signaling pathway through SNHG12/miR-129-5p/MDM4 axis. Our finding provides new insights into the mechanism of ccRCC.

## Data Availability Statement

Publicly available datasets were analyzed in this study. The data of ccRCC patients from TCGA can be found here: https://xena.ucsc.edu.

The data of ccRCC patients from ICGC Data Portal can be downloaded from here: https://dcc.icgc.org/projects/RECA-EU.

Predicted results from LncBase Predicted v.2 and StarBase v2.0 and analyzed results of Pearson correlation analysis between SNHG12 and 1,350 potential target genes were affiliated in Data Sheet 1.

## Author Contributions

XX and CC conceived and designed the project. ZW, DC, and KW performed the experiments and acquired the data. DC analyzed the data. ZW and KW participated in writing the article. All authors read and approved the final version of this manuscript.

### Conflict of Interest

The authors declare that the research was conducted in the absence of any commercial or financial relationships that could be construed as a potential conflict of interest. The reviewer PX declared a shared affiliation, though no other collaboration, with the authors to the handling Editor.
