# Supplementary material for: Long Non-coding RNA SNHG12 Functions as a Competing Endogenous RNA to Regulate MDM4 Expression by Sponging miR-129-5p in Clear Cell Renal Cell Carcinoma
Source: Front Oncol. 2019 Nov 22;9:1260. doi: 10.3389/fonc.2019.01260 (PMC6882951; doi:10.3389/fonc.2019.01260)
Supplement: Supplementary file 1 [file Data_Sheet_1.pdf]

Pearson correlation analysis between SNHG12 and 1,350 potential target genes

LncRNA	target gene	cor	pvalue
SNHG12	CLEC2D	0.683	3.49E-85
SNHG12	WSB1	0.681	1.37E-84
SNHG12	RSRP1	0.667	4.92E-80
SNHG12	RBM33	0.652	3.38E-75
SNHG12	EIF4A1	0.638	3.58E-71
SNHG12	DONSON	0.629	1.06E-68
SNHG12	GIGYF1	0.629	1.47E-68
SNHG12	TIA1	0.611	7.52E-64
SNHG12	ADAT2	0.598	1.97E-60
SNHG12	MDM4	0.585	2.70E-57
SNHG12	NKTR	0.567	3.70E-53
SNHG12	NABP1	0.561	6.46E-52
SNHG12	AL365205.	0.557	4.16E-51
SNHG12	CSNK1E	0.557	4.85E-51
SNHG12	ATG12	0.551	1.05E-49
SNHG12	SRSF11	0.534	2.47E-46
SNHG12	RALGDS	0.533	4.42E-46
SNHG12	YBX3	0.526	9.16E-45
SNHG12	PHF21A	0.525	1.62E-44
SNHG12	CCNL1	0.52	1.30E-43
SNHG12	PNISR	0.518	2.43E-43
SNHG12	UPF3B	0.512	4.49E-42
SNHG12	PLEKHO1	0.498	1.32E-39
SNHG12	ZRANB2	0.492	1.73E-38
SNHG12	SKP2	0.492	1.61E-38
SNHG12	NAGK	0.491	1.87E-38
SNHG12	CREBZF	0.483	4.57E-37
SNHG12	PLCG1	0.479	2.60E-36
SNHG12	CCNF	0.46	2.15E-33
SNHG12	ZNF706	0.456	1.00E-32
SNHG12	BAZ1A	0.45	7.52E-32
SNHG12	TRIP13	0.447	2.78E-31
SNHG12	ITGAE	0.445	5.51E-31
SNHG12	TNRC6A	0.443	8.15E-31
SNHG12	ETV6	0.429	1.02E-28
SNHG12	DLGAP4	0.426	2.34E-28
SNHG12	ZNF587	0.421	1.08E-27
SNHG12	ZNF395	0.421	1.23E-27
SNHG12	MED26	0.42	1.67E-27
SNHG12	MTF2	0.411	3.05E-26
SNHG12	GOLGA3	0.411	3.06E-26
SNHG12	CBLB	0.411	2.44E-26
SNHG12	CNTRL	0.41	3.61E-26
SNHG12	HNRNPA1	0.41	3.46E-26
SNHG12	RGS17	0.409	5.22E-26
SNHG12	FBXW7	0.408	7.42E-26
SNHG12	RUNX1	0.406	1.25E-25
SNHG12	AP3S1	0.403	3.20E-25
SNHG12	DNMT3A	0.402	4.03E-25
SNHG12	TRIO	0.4	7.26E-25
SNHG12	PRR11	0.399	1.08E-24
SNHG12	POGZ	0.397	1.72E-24
SNHG12	RNF217	0.394	4.12E-24
SNHG12	U2AF1	0.393	5.83E-24
SNHG12	CELSR3	0.393	5.63E-24
SNHG12	RBM25	0.392	7.74E-24
SNHG12	ANKRD49	0.39	1.19E-23

SNHG12	LRIG2	0.39	1.30E-23
SNHG12	KIF20A	0.389	1.82E-23
SNHG12	SENP3-EIF	0.386	3.49E-23
SNHG12	AL096711.	0.386	4.14E-23
SNHG12	U2SURP	0.384	6.24E-23
SNHG12	BCL11B	0.381	1.47E-22
SNHG12	PBX2	0.376	5.47E-22
SNHG12	GIT1	0.374	1.07E-21
SNHG12	TIAL1	0.373	1.36E-21
SNHG12	NMI	0.371	2.14E-21
SNHG12	ORC2	0.367	7.11E-21
SNHG12	SCMH1	0.367	6.46E-21
SNHG12	ENOSF1	0.366	9.26E-21
SNHG12	PABPC1	0.366	9.10E-21
SNHG12	SAMD10	0.366	8.92E-21
SNHG12	CPSF7	0.364	1.38E-20
SNHG12	ZNF532	0.36	3.90E-20
SNHG12	SLAMF7	0.357	7.84E-20
SNHG12	RBM26	0.356	1.13E-19
SNHG12	E2F7	0.356	1.19E-19
SNHG12	GATAD1	0.356	1.18E-19
SNHG12	BRCA2	0.354	1.68E-19
SNHG12	HOXA3	0.351	3.65E-19
SNHG12	MATR3	0.348	8.24E-19
SNHG12	PHF12	0.347	9.82E-19
SNHG12	USF2	0.346	1.13E-18
SNHG12	PPP1R3B	0.343	2.59E-18
SNHG12	UBN2	0.341	4.71E-18
SNHG12	CREB5	0.339	6.31E-18
SNHG12	HELLS	0.337	1.15E-17
SNHG12	RASSF5	0.335	1.57E-17
SNHG12	SLC39A14	0.334	2.39E-17
SNHG12	ZDHHC17	0.334	1.98E-17
SNHG12	PRDM1	0.333	2.74E-17
SNHG12	IRF1	0.328	9.00E-17
SNHG12	RNF213	0.326	1.46E-16
SNHG12	TP53INP1	0.325	1.63E-16
SNHG12	PDCD6	0.324	1.90E-16
SNHG12	TYMS	0.324	1.92E-16
SNHG12	BICD1	0.323	2.51E-16
SNHG12	KIF18A	0.323	2.70E-16
SNHG12	METTL23	0.322	3.35E-16
SNHG12	TGM2	0.32	4.97E-16
SNHG12	IRF4	0.316	1.24E-15
SNHG12	CNEP1R1	0.315	1.51E-15
SNHG12	GAN	0.314	1.80E-15
SNHG12	P4HA2	0.312	2.82E-15
SNHG12	ARL6IP6	0.311	3.96E-15
SNHG12	ZMYND8	0.311	3.35E-15
SNHG12	U2AF1L5	0.309	5.60E-15
SNHG12	HERC4	0.307	8.31E-15
SNHG12	RBM41	0.307	9.21E-15
SNHG12	KDM2B	0.305	1.19E-14
SNHG12	CCDC88A	0.301	2.97E-14
SNHG12	AGO3	0.301	3.00E-14
SNHG12	CCAR1	0.3	3.76E-14
SNHG12	ATAD2B	0.3	3.65E-14
SNHG12	QSOX2	0.298	4.88E-14

SNHG12	ZNF385A	0.298	5.60E-14
SNHG12	PMP22	0.297	6.89E-14
SNHG12	SRSF7	0.296	7.58E-14
SNHG12	MAML1	0.295	1.05E-13
SNHG12	PON2	0.295	9.79E-14
SNHG12	BASP1	0.291	2.01E-13
SNHG12	RBM3	0.289	3.52E-13
SNHG12	ARPC5	0.285	7.52E-13
SNHG12	ZNF280C	0.285	6.76E-13
SNHG12	RAD23A	0.284	8.05E-13
SNHG12	KCTD3	0.284	8.61E-13
SNHG12	KIAA0408	0.281	1.63E-12
SNHG12	SPATS2	0.28	1.99E-12
SNHG12	COL1A1	0.28	1.91E-12
SNHG12	RSRC2	0.279	2.09E-12
SNHG12	BCL2L12	0.279	2.12E-12
SNHG12	CDK1	0.279	2.39E-12
SNHG12	DUSP18	0.277	3.01E-12
SNHG12	RPS6KB1	0.272	8.03E-12
SNHG12	TTK	0.272	8.10E-12
SNHG12	POLR1D	0.272	7.80E-12
SNHG12	MEX3B	0.271	1.04E-11
SNHG12	ELL2	0.271	9.82E-12
SNHG12	PTPN1	0.268	1.65E-11
SNHG12	PMEPA1	0.267	1.99E-11
SNHG12	TAOK2	0.266	2.47E-11
SNHG12	CDK5R1	0.265	2.77E-11
SNHG12	DTL	0.265	2.69E-11
SNHG12	STK17B	0.264	3.09E-11
SNHG12	ZNF605	0.264	3.10E-11
SNHG12	TRIM14	0.264	3.64E-11
SNHG12	CBFA2T3	0.256	1.43E-10
SNHG12	FNBP1	0.255	1.48E-10
SNHG12	DYRK2	0.254	1.75E-10
SNHG12	DEGS1	0.254	1.96E-10
SNHG12	JARID2	0.251	3.29E-10
SNHG12	CCR7	0.251	2.95E-10
SNHG12	PWWP2A	0.25	3.86E-10
SNHG12	XPO1	0.249	4.09E-10
SNHG12	INO80D	0.249	4.57E-10
SNHG12	ASAP1	0.248	5.10E-10
SNHG12	AHDC1	0.247	6.13E-10
SNHG12	CMIP	0.245	7.82E-10
SNHG12	ACTN1	0.244	9.26E-10
SNHG12	EPC1	0.243	1.26E-09
SNHG12	SCHIP1	0.24	1.84E-09
SNHG12	MARCKS	0.24	1.82E-09
SNHG12	RUBCN	0.239	2.13E-09
SNHG12	ABL2	0.239	2.27E-09
SNHG12	SALL4	0.239	2.35E-09
SNHG12	CPSF6	0.237	3.18E-09
SNHG12	HERPUD2	0.237	2.86E-09
SNHG12	RND3	0.236	3.32E-09
SNHG12	BCL11A	0.235	4.33E-09
SNHG12	MAF	0.233	5.37E-09
SNHG12	TMX3	0.233	5.25E-09
SNHG12	KDM2A	0.229	1.01E-08
SNHG12	SLC35A1	0.226	1.51E-08

SNHG12	NFKBIA	0.225	2.02E-08
SNHG12	KANSL1	0.225	1.81E-08
SNHG12	TXNDC12	0.224	2.12E-08
SNHG12	TMEM43	0.224	2.18E-08
SNHG12	FIGN	0.221	3.46E-08
SNHG12	ZNF37A	0.221	3.49E-08
SNHG12	AC005520	0.22	3.91E-08
SNHG12	SH2B3	0.219	4.71E-08
SNHG12	ZEB2	0.218	5.29E-08
SNHG12	OAS2	0.217	5.85E-08
SNHG12	SSH2	0.217	6.36E-08
SNHG12	MAFB	0.215	8.04E-08
SNHG12	ZC3H11A	0.214	9.49E-08
SNHG12	ZNF431	0.213	1.08E-07
SNHG12	RPL14	0.213	1.05E-07
SNHG12	MRPL22	0.213	1.11E-07
SNHG12	HNRNPA1	0.212	1.29E-07
SNHG12	HIC2	0.211	1.33E-07
SNHG12	BAZ2A	0.211	1.44E-07
SNHG12	KHSRP	0.211	1.35E-07
SNHG12	ZNF708	0.211	1.33E-07
SNHG12	DLX2	0.21	1.71E-07
SNHG12	UBA6	0.209	1.82E-07
SNHG12	SH3KBP1	0.206	2.76E-07
SNHG12	NREP	0.206	2.71E-07
SNHG12	ATF5	0.205	3.08E-07
SNHG12	ATF7	0.205	3.10E-07
SNHG12	PTMA	0.205	3.10E-07
SNHG12	RNF115	0.204	3.89E-07
SNHG12	SYBU	0.203	4.16E-07
SNHG12	POLH	0.197	8.80E-07
SNHG12	SP1	0.196	1.06E-06
SNHG12	TANK	0.194	1.38E-06
SNHG12	CHD1	0.194	1.39E-06
SNHG12	POM121C	0.193	1.62E-06
SNHG12	ZIC2	0.192	1.72E-06
SNHG12	SOX11	0.19	2.35E-06
SNHG12	ABHD18	0.19	2.30E-06
SNHG12	SOD2	0.19	2.12E-06
SNHG12	LPGAT1	0.189	2.62E-06
SNHG12	FOXK1	0.188	2.85E-06
SNHG12	WTAP	0.187	3.27E-06
SNHG12	STOX2	0.186	3.61E-06
SNHG12	FYN	0.181	7.13E-06
SNHG12	HMGA2	0.18	7.30E-06
SNHG12	SKI	0.18	7.20E-06
SNHG12	IRS2	0.18	8.02E-06
SNHG12	CELF1	0.18	7.69E-06
SNHG12	OTX1	0.179	8.82E-06
SNHG12	HOOK3	0.174	1.46E-05
SNHG12	C14orf28	0.174	1.56E-05
SNHG12	PSMB7	0.174	1.55E-05
SNHG12	DDIAS	0.172	1.99E-05
SNHG12	SMG1	0.171	2.12E-05
SNHG12	PITX1	0.171	2.16E-05
SNHG12	KRIT1	0.17	2.33E-05
SNHG12	SCAF4	0.169	2.65E-05
SNHG12	MKNK2	0.168	2.90E-05

SNHG12	SS18L1	0.166	3.64E-05
SNHG12	CDK13	0.166	3.63E-05
SNHG12	KLF6	0.165	4.37E-05
SNHG12	PLD3	0.165	3.96E-05
SNHG12	ZSWIM6	0.164	4.62E-05
SNHG12	COL4A1	0.163	5.32E-05
SNHG12	LMNA	0.163	5.03E-05
SNHG12	RNF125	0.163	5.40E-05
SNHG12	GSE1	0.163	5.41E-05
SNHG12	GDF11	0.161	6.15E-05
SNHG12	DUSP14	0.16	7.27E-05
SNHG12	MXD1	0.159	7.95E-05
SNHG12	KMT2D	0.157	9.39E-05
SNHG12	GPX8	0.154	0.000128
SNHG12	GGNBP2	0.153	0.000144
SNHG12	AKAP13	0.153	0.000142
SNHG12	NCK1	0.152	0.000158
SNHG12	REV1	0.151	0.000175
SNHG12	FHL1	0.15	0.00019
SNHG12	ZNF410	0.15	0.000191
SNHG12	SERTAD2	0.149	0.00022
SNHG12	ZNF740	0.148	0.000235
SNHG12	CD47	0.147	0.000269
SNHG12	SLC20A1	0.146	0.000307
SNHG12	MORC3	0.145	0.000314
SNHG12	MIS12	0.145	0.000312
SNHG12	COL11A1	0.145	0.000315
SNHG12	GPATCH2	0.144	0.00036
SNHG12	EGFR	0.143	0.000398
SNHG12	YWHAH	0.143	0.000389
SNHG12	TMEM154	0.143	0.000384
SNHG12	PLEKHA1	0.142	0.000413
SNHG12	POU4F1	0.141	0.000478
SNHG12	DPY19L1	0.14	0.000508
SNHG12	KMT2E	0.138	0.000641
SNHG12	PELI1	0.138	0.000604
SNHG12	MBNL1	0.138	0.000626
SNHG12	CCND1	0.137	0.000679
SNHG12	ABCC5	0.137	0.000658
SNHG12	STRIP1	0.137	0.000658
SNHG12	CPE	0.135	0.000818
SNHG12	ATP8B2	0.134	0.000928
SNHG12	HMGXB4	0.134	0.000927
SNHG12	E2F3	0.133	0.000942
SNHG12	CEBPZOS	0.132	0.001117
SNHG12	HNRNPUL	0.13	0.001281
SNHG12	PHIP	0.129	0.001346
SNHG12	ZNF638	0.129	0.001402
SNHG12	TBL1XR1	0.129	0.00144
SNHG12	TPM3	0.127	0.001668
SNHG12	MARCKSL1	0.127	0.001687
SNHG12	NR3C1	0.127	0.001662
SNHG12	MEIS1	0.126	0.001798
SNHG12	EIF6	0.126	0.00185
SNHG12	ADARB1	0.125	0.002041
SNHG12	CBX4	0.125	0.002022
SNHG12	SHCBP1	0.124	0.002175
SNHG12	TGIF2	0.124	0.002201

SNHG12	COL3A1	0.124	0.002099
SNHG12	LEPROTL1	0.124	0.002091
SNHG12	IDS	0.124	0.002071
SNHG12	POC1B-GA	0.123	0.002302
SNHG12	BRWD1	0.123	0.002349
SNHG12	NFX1	0.122	0.002578
SNHG12	FGFR1OP2	0.121	0.002733
SNHG12	UBAP2L	0.12	0.003053
SNHG12	ZNF791	0.12	0.003052
SNHG12	C12orf43	0.12	0.002977
SNHG12	FNDC3B	0.119	0.003259
SNHG12	POU3F2	0.119	0.003174
SNHG12	MYADM	0.119	0.003255
SNHG12	RORA	0.118	0.003406
SNHG12	UBE2Z	0.118	0.00353
SNHG12	ENTPD4	0.118	0.003398
SNHG12	EXOSC3	0.117	0.003636
SNHG12	ANP32B	0.117	0.003689
SNHG12	TXNDC15	0.117	0.003889
SNHG12	MEF2D	0.117	0.003702
SNHG12	DCLRE1B	0.116	0.004245
SNHG12	FNIP1	0.116	0.003976
SNHG12	STIM2	0.116	0.004095
SNHG12	SMCHD1	0.115	0.004318
SNHG12	TSR1	0.115	0.004296
SNHG12	STK39	0.114	0.004672
SNHG12	LRIG3	0.113	0.00503
SNHG12	IRF2	0.112	0.005688
SNHG12	NUP58	0.112	0.005554
SNHG12	ZNF703	0.112	0.005534
SNHG12	PPP1R12A	0.111	0.005861
SNHG12	ZMYM2	0.111	0.005916
SNHG12	PROK2	0.109	0.007072
SNHG12	CDH2	0.108	0.007727
SNHG12	FAM208B	0.107	0.007987
SNHG12	TIPARP	0.107	0.008074
SNHG12	ZNF695	0.107	0.007855
SNHG12	TUBGCP3	0.106	0.008802
SNHG12	TET2	0.105	0.009198
SNHG12	PIGC	0.105	0.009427
SNHG12	CCP110	0.105	0.009257
SNHG12	IKZF2	0.104	0.010041
SNHG12	ARID4B	0.103	0.010905
SNHG12	FOXG1	0.102	0.011501
SNHG12	MAP3K20	0.102	0.011784
SNHG12	TTBK2	0.102	0.011831
SNHG12	DDX3Y	0.102	0.011891
SNHG12	DKC1	0.101	0.012163
SNHG12	HOXC13	0.101	0.012805
SNHG12	KEAP1	0.101	0.012093
SNHG12	MBD4	0.1	0.013002
SNHG12	SSH1	0.099	0.014483
SNHG12	CALU	0.096	0.017294
SNHG12	ELF2	0.096	0.017347
SNHG12	WBP2	0.095	0.019321
SNHG12	FOXA1	0.093	0.021328
SNHG12	PGM2L1	0.093	0.021395
SNHG12	MIDN	0.092	0.023491

SNHG12	NDUFV3	0.091	0.024296
SNHG12	RC3H1	0.09	0.025536
SNHG12	MTMR4	0.089	0.028586
SNHG12	PHLDA1	0.088	0.029861
SNHG12	ETS1	0.086	0.033506
SNHG12	ATXN7	0.086	0.033851
SNHG12	CNIH4	0.084	0.037813
SNHG12	TANC2	0.083	0.041065
SNHG12	KPNB1	0.083	0.040865
SNHG12	KDELC2	0.082	0.042642
SNHG12	CAMK4	0.08	0.048716
SNHG12	ANKRD12	0.08	0.049167
SNHG12	UFM1	0.079	0.050925
SNHG12	JAZF1	0.079	0.050664
SNHG12	PITPNB	0.079	0.049975
SNHG12	CASP6	0.078	0.055464
SNHG12	SLC31A2	0.077	0.057642
SNHG12	KIF2A	0.077	0.057122
SNHG12	RBPJ	0.077	0.05734
SNHG12	CARM1	0.076	0.059174
SNHG12	RBMS1	0.075	0.062192
SNHG12	NRXN3	0.073	0.072375
SNHG12	GLCCI1	0.073	0.071211
SNHG12	NEDD4	0.072	0.074914
SNHG12	TNFSF10	0.071	0.078535
SNHG12	CHML	0.071	0.078638
SNHG12	CHD7	0.07	0.082224
SNHG12	LARP1	0.069	0.086675
SNHG12	HECA	0.068	0.094814
SNHG12	MID1	0.068	0.092038
SNHG12	GALNT1	0.067	0.095913
SNHG12	PSMG2	0.067	0.096647
SNHG12	HDGF	0.067	0.099106
SNHG12	AMMECR1	0.066	0.105079
SNHG12	BAZ1B	0.066	0.103181
SNHG12	MFSD8	0.066	0.10152
SNHG12	BMP2K	0.065	0.106387
SNHG12	SLC25A36	0.065	0.105961
SNHG12	ZFHX4	0.065	0.106033
SNHG12	ABI3BP	0.064	0.113481
SNHG12	SNAPC1	0.063	0.118244
SNHG12	KMT2A	0.063	0.117773
SNHG12	NFIB	0.063	0.118601
SNHG12	CEBPG	0.063	0.117678
SNHG12	BICD2	0.063	0.120573
SNHG12	WDR26	0.061	0.133927
SNHG12	AC026786	0.06	0.135621
SNHG12	OXR1	0.06	0.141255
SNHG12	PAPOLG	0.06	0.139814
SNHG12	IRGQ	0.059	0.143153
SNHG12	POU2F1	0.059	0.142372
SNHG12	CNDP2	0.059	0.145625
SNHG12	ELMSAN1	0.057	0.159691
SNHG12	TCF4	0.056	0.16929
SNHG12	SNRPC	0.056	0.167437
SNHG12	FAM160B1	0.055	0.17452
SNHG12	NR2C2	0.055	0.174916
SNHG12	GLUL	0.055	0.176729

SNHG12	SMNDC1	0.054	0.180346
SNHG12	ONECUT2	0.053	0.194691
SNHG12	ZBTB34	0.053	0.190348
SNHG12	LIPA	0.052	0.199921
SNHG12	GOLT1B	0.052	0.203492
SNHG12	PLEKHA2	0.051	0.207058
SNHG12	IFRD2	0.05	0.216027
SNHG12	ING3	0.05	0.220883
SNHG12	YWHAZ	0.049	0.222844
SNHG12	ELK4	0.049	0.225607
SNHG12	ZNF623	0.048	0.236099
SNHG12	RAC1	0.048	0.233132
SNHG12	MAST4	0.048	0.234515
SNHG12	NFIA	0.047	0.248569
SNHG12	JDP2	0.047	0.24708
SNHG12	MARK2	0.047	0.248337
SNHG12	ZBTB8A	0.047	0.242871
SNHG12	SLC7A2	0.046	0.259468
SNHG12	ZBTB7A	0.046	0.25455
SNHG12	RAI14	0.045	0.263229
SNHG12	DGKE	0.044	0.282782
SNHG12	CEP350	0.044	0.28014
SNHG12	SOX9	0.043	0.284413
SNHG12	NBN	0.043	0.287512
SNHG12	TSPAN13	0.043	0.292052
SNHG12	MAP3K2	0.043	0.293361
SNHG12	SGK3	0.043	0.286247
SNHG12	LIMS1	0.042	0.296
SNHG12	NPEPPS	0.041	0.313597
SNHG12	MAP1B	0.04	0.317706
SNHG12	TMA16	0.04	0.325513
SNHG12	TMEM250	0.039	0.341516
SNHG12	TTPAL	0.036	0.370395
SNHG12	ZBTB43	0.036	0.372511
SNHG12	SLC35E1	0.036	0.380744
SNHG12	SPOP	0.036	0.375737
SNHG12	RFX7	0.035	0.392821
SNHG12	AKAP10	0.035	0.387004
SNHG12	HNRNPH3	0.035	0.388279
SNHG12	HNRNPA3	0.035	0.385632
SNHG12	GPBP1	0.035	0.385038
SNHG12	SUMO2	0.034	0.403358
SNHG12	RSL24D1	0.034	0.405716
SNHG12	HMGB1	0.031	0.440541
SNHG12	ARHGAP3:	0.031	0.440549
SNHG12	TMPO	0.031	0.444978
SNHG12	TXN	0.03	0.455873
SNHG12	KPNA5	0.03	0.457553
SNHG12	R3HDM1	0.029	0.47846
SNHG12	CKAP4	0.028	0.495303
SNHG12	TMEFF1	0.028	0.483211
SNHG12	FAR1	0.027	0.509702
SNHG12	EIF4A2	0.027	0.507074
SNHG12	ATP13A3	0.026	0.513665
SNHG12	EMP2	0.026	0.515866
SNHG12	LRIG1	0.026	0.515184
SNHG12	MED13L	0.025	0.54063
SNHG12	ETV1	0.025	0.541804

SNHG12	ELK3	0.024	0.553501
SNHG12	MTR	0.024	0.547546
SNHG12	TNRC6C	0.024	0.557273
SNHG12	SLC10A7	0.022	0.591647
SNHG12	DNAJC24	0.021	0.612813
SNHG12	BCAP29	0.02	0.616797
SNHG12	C11orf49	0.019	0.647148
SNHG12	PGK1	0.019	0.642915
SNHG12	TCF20	0.019	0.635143
SNHG12	ZFP36L1	0.019	0.645039
SNHG12	KBTBD2	0.018	0.654705
SNHG12	FKBP14	0.018	0.652969
SNHG12	CCDC170	0.017	0.683934
SNHG12	CREBBP	0.017	0.670875
SNHG12	VPS13A	0.016	0.699266
SNHG12	MAP3K7	0.016	0.694231
SNHG12	ZFAND5	0.016	0.687412
SNHG12	FBXO45	0.015	0.702623
SNHG12	KIAA1586	0.014	0.722509
SNHG12	CCDC6	0.014	0.734105
SNHG12	TRA2B	0.013	0.757091
SNHG12	ADAMTS5	0.011	0.78821
SNHG12	SLC7A1	0.011	0.782795
SNHG12	GNA12	0.011	0.779148
SNHG12	GATA6	0.01	0.797804
SNHG12	GATAD2B	0.009	0.833788
SNHG12	EPHA7	0.009	0.831742
SNHG12	MOSPD2	0.008	0.839005
SNHG12	CCDC43	0.008	0.834987
SNHG12	ETS2	0.007	0.870801
SNHG12	ZNF451	0.006	0.879149
SNHG12	TNRC6B	0.005	0.895769
SNHG12	F3	0.003	0.93949
SNHG12	KCNK1	0.003	0.945895
SNHG12	PURB	0.002	0.958102
SNHG12	NUFIP2	0.002	0.967023
SNHG12	LRR1	0	0.995042
SNHG12	NUP50	0	0.990454
SNHG12	AK2	0	0.991588
SNHG12	ZNRF1	-0.001	0.972982
SNHG12	CAST	-0.001	0.974275
SNHG12	RASA1	-0.002	0.968839
SNHG12	SRSF3	-0.003	0.937204
SNHG12	ZNF280B	-0.004	0.912214
SNHG12	CTDSPL2	-0.005	0.897598
SNHG12	PCOLCE2	-0.005	0.893083
SNHG12	DDX50	-0.007	0.855582
SNHG12	ARID4A	-0.007	0.867282
SNHG12	MSANTD3	-0.007	0.86278
SNHG12	ACER3	-0.009	0.818797
SNHG12	RERE	-0.011	0.784164
SNHG12	PBX3	-0.011	0.782318
SNHG12	PSMD12	-0.011	0.793932
SNHG12	KLHL20	-0.011	0.785821
SNHG12	S1PR3	-0.012	0.773345
SNHG12	KCTD20	-0.012	0.773736
SNHG12	VPS45	-0.012	0.770036
SNHG12	ZBTB5	-0.013	0.751766

SNHG12	DESI2	-0.014	0.724603
SNHG12	KMT2C	-0.014	0.732224
SNHG12	SEC23A	-0.015	0.707647
SNHG12	CDK6	-0.015	0.705973
SNHG12	SGTB	-0.015	0.717367
SNHG12	ZNF503	-0.016	0.693883
SNHG12	SESN1	-0.016	0.692465
SNHG12	KHDRBS1	-0.016	0.696937
SNHG12	POLD3	-0.016	0.698078
SNHG12	NKRF	-0.017	0.674674
SNHG12	N4BP2	-0.017	0.675252
SNHG12	USP42	-0.018	0.649585
SNHG12	PHC3	-0.018	0.651514
SNHG12	GALNT7	-0.019	0.634708
SNHG12	SEC61A1	-0.02	0.62365
SNHG12	ZKSCAN1	-0.02	0.615423
SNHG12	ARID2	-0.021	0.596011
SNHG12	FOXD1	-0.021	0.604607
SNHG12	CDK12	-0.022	0.592062
SNHG12	YWHAG	-0.022	0.579741
SNHG12	PCBP2	-0.022	0.585197
SNHG12	SP3	-0.023	0.573883
SNHG12	L3MBTL3	-0.023	0.571391
SNHG12	ZNF281	-0.023	0.572728
SNHG12	ROBO1	-0.023	0.564718
SNHG12	BLOC1S6	-0.024	0.547048
SNHG12	TMEM126I	-0.024	0.56038
SNHG12	ESR1	-0.026	0.515871
SNHG12	STAT5B	-0.026	0.526683
SNHG12	SOAT1	-0.027	0.508131
SNHG12	TXNDC5	-0.027	0.500427
SNHG12	LRRC8A	-0.028	0.492606
SNHG12	SPIN4	-0.03	0.458756
SNHG12	ACTR3	-0.031	0.442301
SNHG12	FRYL	-0.033	0.414723
SNHG12	EEA1	-0.033	0.412879
SNHG12	THADA	-0.033	0.415874
SNHG12	CFAP20	-0.034	0.395553
SNHG12	RAB22A	-0.034	0.401396
SNHG12	BOLA3	-0.034	0.400581
SNHG12	PTX3	-0.035	0.38537
SNHG12	ASPH	-0.035	0.382126
SNHG12	PRDM2	-0.036	0.375422
SNHG12	C6orf62	-0.036	0.369739
SNHG12	MLEC	-0.037	0.355508
SNHG12	YOD1	-0.038	0.349143
SNHG12	NUMB	-0.038	0.346245
SNHG12	RBFOX2	-0.038	0.348442
SNHG12	SNAPC3	-0.039	0.33567
SNHG12	BAZ2B	-0.04	0.328659
SNHG12	PHF20	-0.04	0.326014
SNHG12	ERG	-0.041	0.313574
SNHG12	PERP	-0.041	0.313651
SNHG12	KLHL28	-0.042	0.302193
SNHG12	GXYLT1	-0.042	0.301999
SNHG12	DENND6A	-0.043	0.29268
SNHG12	LYRM1	-0.043	0.283809
SNHG12	MED14	-0.043	0.287553

SNHG12	MTX3	-0.043	0.283767
SNHG12	PANK2	-0.046	0.257176
SNHG12	KIF3A	-0.046	0.259002
SNHG12	C9orf40	-0.047	0.250165
SNHG12	WNT5A	-0.048	0.236903
SNHG12	FBN2	-0.048	0.240536
SNHG12	SERINC3	-0.048	0.235914
SNHG12	STX3	-0.05	0.218106
SNHG12	LBR	-0.05	0.220115
SNHG12	ACAP2	-0.05	0.216443
SNHG12	THOC7	-0.05	0.216744
SNHG12	ARNT	-0.05	0.216649
SNHG12	DMD	-0.05	0.214147
SNHG12	PLAGL2	-0.052	0.195399
SNHG12	ARL8A	-0.053	0.194946
SNHG12	CENPQ	-0.053	0.191823
SNHG12	BPTF	-0.053	0.191464
SNHG12	MSN	-0.056	0.165376
SNHG12	SIPA1L1	-0.056	0.163302
SNHG12	TPBG	-0.056	0.165951
SNHG12	FAM217B	-0.057	0.156243
SNHG12	ARL5B	-0.058	0.153602
SNHG12	STYX	-0.058	0.154844
SNHG12	ZNF91	-0.059	0.146939
SNHG12	SIKE1	-0.059	0.14712
SNHG12	PHF8	-0.06	0.140617
SNHG12	ANAPC16	-0.061	0.133874
SNHG12	SEMA3C	-0.061	0.132958
SNHG12	BTBD3	-0.061	0.132956
SNHG12	NFIC	-0.062	0.128725
SNHG12	RCOR1	-0.062	0.122996
SNHG12	WWTR1	-0.063	0.119821
SNHG12	DYRK1A	-0.063	0.119686
SNHG12	EPAS1	-0.064	0.112249
SNHG12	NIPBL	-0.064	0.11289
SNHG12	BCL7B	-0.066	0.105472
SNHG12	MKRN1	-0.067	0.096229
SNHG12	VPS53	-0.067	0.098535
SNHG12	WAC	-0.067	0.096676
SNHG12	MACO1	-0.067	0.10028
SNHG12	EIF4G3	-0.068	0.094536
SNHG12	ZBTB20	-0.068	0.094502
SNHG12	ITPRIPL2	-0.069	0.088852
SNHG12	KIF5C	-0.069	0.089336
SNHG12	TTC3	-0.069	0.086396
SNHG12	IGF2BP2	-0.069	0.087712
SNHG12	TRIM71	-0.07	0.085301
SNHG12	CACUL1	-0.07	0.084224
SNHG12	OTUD6B	-0.071	0.077536
SNHG12	ZFP91-CN	-0.071	0.080867
SNHG12	EXT1	-0.072	0.076867
SNHG12	DNAJC12	-0.072	0.076022
SNHG12	SOX4	-0.072	0.075068
SNHG12	KIAA1429	-0.073	0.071716
SNHG12	DCTN4	-0.074	0.066657
SNHG12	UBE2J1	-0.074	0.067679
SNHG12	EPM2AIP1	-0.075	0.065143
SNHG12	SARAF	-0.075	0.063159

SNHG12	IFT74	-0.076	0.060816
SNHG12	PTEN	-0.077	0.056779
SNHG12	FAM13B	-0.078	0.053353
SNHG12	SBNO1	-0.078	0.05344
SNHG12	SNRNP27	-0.079	0.050281
SNHG12	GPRC5A	-0.079	0.049786
SNHG12	CTTNBP2N	-0.079	0.052358
SNHG12	TC2N	-0.079	0.049595
SNHG12	RBM4	-0.079	0.051168
SNHG12	METTL21A	-0.08	0.047314
SNHG12	EDEM3	-0.082	0.042851
SNHG12	QKI	-0.082	0.042059
SNHG12	FOXO3	-0.083	0.040096
SNHG12	SPIRE1	-0.083	0.040128
SNHG12	RBM27	-0.083	0.039529
SNHG12	SNX16	-0.084	0.038772
SNHG12	ZNF551	-0.084	0.038641
SNHG12	EIF4B	-0.084	0.038356
SNHG12	GOLGA5	-0.084	0.038561
SNHG12	SETDB2	-0.085	0.035849
SNHG12	CDC27	-0.085	0.036355
SNHG12	ZNF720	-0.086	0.034365
SNHG12	PTP4A2	-0.086	0.034001
SNHG12	AJUBA	-0.086	0.032702
SNHG12	LPP	-0.087	0.031813
SNHG12	UBE2A	-0.087	0.03199
SNHG12	CREM	-0.087	0.032379
SNHG12	SPPL3	-0.088	0.029048
SNHG12	MIER1	-0.089	0.027961
SNHG12	LNPEP	-0.09	0.026619
SNHG12	ZFP36L2	-0.09	0.025874
SNHG12	UBE2D1	-0.09	0.026208
SNHG12	PKN2	-0.09	0.025298
SNHG12	IGFBP7	-0.09	0.026422
SNHG12	LCOR	-0.091	0.024897
SNHG12	PDS5A	-0.092	0.023591
SNHG12	UBXN7	-0.092	0.022632
SNHG12	EIF5B	-0.092	0.022559
SNHG12	GAPVD1	-0.093	0.021813
SNHG12	PKP2	-0.093	0.021764
SNHG12	MYLIP	-0.093	0.021676
SNHG12	NIP7	-0.094	0.019666
SNHG12	BICC1	-0.094	0.020487
SNHG12	BCL10	-0.094	0.020069
SNHG12	LMTK2	-0.095	0.019393
SNHG12	H3F3A	-0.096	0.017781
SNHG12	DEK	-0.096	0.017473
SNHG12	FOXP4	-0.097	0.016802
SNHG12	UBE2V2	-0.098	0.014922
SNHG12	DPY19L3	-0.098	0.01534
SNHG12	HOXA5	-0.098	0.015757
SNHG12	FST	-0.099	0.013922
SNHG12	TMEM123	-0.101	0.012144
SNHG12	TRPS1	-0.102	0.011589
SNHG12	INTS6	-0.102	0.011475
SNHG12	ZBTB18	-0.102	0.011608
SNHG12	VPS13B	-0.104	0.010251
SNHG12	EIF2AK2	-0.104	0.009814

SNHG12	RAD1	-0.105	0.00951
SNHG12	PHF3	-0.105	0.00946
SNHG12	ROCK1	-0.105	0.009577
SNHG12	ARL6IP5	-0.106	0.008853
SNHG12	ZNF480	-0.106	0.008691
SNHG12	ELAVL1	-0.107	0.007873
SNHG12	SNTB2	-0.109	0.006856
SNHG12	CLIP1	-0.109	0.007072
SNHG12	SHANK2	-0.11	0.006314
SNHG12	SETD7	-0.11	0.006458
SNHG12	CAMK2D	-0.11	0.006394
SNHG12	PRKCD	-0.111	0.006181
SNHG12	ZFHX3	-0.111	0.005839
SNHG12	AMOTL2	-0.114	0.004778
SNHG12	FCHO2	-0.114	0.00479
SNHG12	RPL7L1	-0.117	0.003776
SNHG12	EMP1	-0.117	0.00392
SNHG12	KDM5A	-0.118	0.003562
SNHG12	LATS1	-0.118	0.003491
SNHG12	PIK3R1	-0.118	0.003588
SNHG12	SPOCK1	-0.119	0.003331
SNHG12	ZBED6	-0.119	0.003214
SNHG12	NMD3	-0.119	0.003093
SNHG12	DDX18	-0.119	0.003173
SNHG12	DAB2IP	-0.121	0.002683
SNHG12	CNBP	-0.121	0.002656
SNHG12	UTP23	-0.121	0.002844
SNHG12	GCLM	-0.122	0.002505
SNHG12	ADAR	-0.122	0.002549
SNHG12	USP37	-0.122	0.002561
SNHG12	DDX21	-0.122	0.002421
SNHG12	USP47	-0.122	0.002496
SNHG12	DST	-0.124	0.002171
SNHG12	GOLGB1	-0.125	0.001997
SNHG12	OTUD7B	-0.125	0.001932
SNHG12	PPP6R3	-0.128	0.00153
SNHG12	DLC1	-0.128	0.001492
SNHG12	NUP205	-0.129	0.001426
SNHG12	CSNK1G1	-0.129	0.001388
SNHG12	RNF2	-0.129	0.001388
SNHG12	SWAP70	-0.13	0.001296
SNHG12	CDC42SE2	-0.13	0.001311
SNHG12	CHD9	-0.131	0.001157
SNHG12	TSHZ1	-0.133	0.000971
SNHG12	PIKFYVE	-0.134	0.000913
SNHG12	GCC2	-0.134	0.00088
SNHG12	MAP3K1	-0.134	0.000923
SNHG12	TULP4	-0.134	0.000864
SNHG12	ZMIZ1	-0.135	0.000808
SNHG12	PGRMC2	-0.136	0.000724
SNHG12	PTGES3	-0.136	0.000764
SNHG12	YIPF5	-0.137	0.000663
SNHG12	ABCE1	-0.137	0.000681
SNHG12	CNOT4	-0.138	0.000617
SNHG12	PHACTR2	-0.138	0.000636
SNHG12	C11orf58	-0.138	0.000638
SNHG12	B4GALT1	-0.139	0.000575
SNHG12	SNX3	-0.14	0.000522

SNHG12	ROCK2	-0.14	0.000537
SNHG12	TRAM1	-0.14	0.000497
SNHG12	HAUS2	-0.141	0.000485
SNHG12	KCTD10	-0.141	0.000478
SNHG12	KLF13	-0.141	0.000477
SNHG12	UBE4B	-0.142	0.00045
SNHG12	PTAR1	-0.142	0.000447
SNHG12	SNX27	-0.143	0.000389
SNHG12	RB1CC1	-0.143	0.000384
SNHG12	RTF1	-0.143	0.000407
SNHG12	RSBN1	-0.144	0.000359
SNHG12	STAT3	-0.144	0.000369
SNHG12	CHD6	-0.146	0.000283
SNHG12	PCNP	-0.146	0.000296
SNHG12	KMT5B	-0.148	0.000242
SNHG12	ZNF112	-0.149	0.000222
SNHG12	VKORC1L1	-0.15	0.000203
SNHG12	WIPF2	-0.15	0.000206
SNHG12	ADAM10	-0.15	0.000202
SNHG12	MBNL2	-0.15	0.000202
SNHG12	MYO9A	-0.151	0.000187
SNHG12	CAMTA1	-0.151	0.000184
SNHG12	SPCS3	-0.151	0.000184
SNHG12	NUP153	-0.151	0.000175
SNHG12	G3BP1	-0.152	0.000164
SNHG12	DFFA	-0.153	0.000148
SNHG12	ZBTB41	-0.153	0.000153
SNHG12	CCNYL1	-0.153	0.000148
SNHG12	STRN3	-0.154	0.000136
SNHG12	CSNK1A1	-0.154	0.000133
SNHG12	AFF1	-0.154	0.000129
SNHG12	MBLAC2	-0.156	0.000112
SNHG12	MEF2A	-0.156	0.000111
SNHG12	FOXP1	-0.156	0.000106
SNHG12	ICE1	-0.156	0.000113
SNHG12	PRRC1	-0.156	0.00011
SNHG12	ARIH1	-0.157	9.43E-05
SNHG12	EP300	-0.158	9.20E-05
SNHG12	DICER1	-0.159	8.10E-05
SNHG12	LSM12	-0.159	7.70E-05
SNHG12	HNRNPA0	-0.159	8.28E-05
SNHG12	IPCEF1	-0.16	7.25E-05
SNHG12	TOMM20	-0.16	7.19E-05
SNHG12	KAT6B	-0.161	6.29E-05
SNHG12	ESD	-0.162	5.82E-05
SNHG12	LIN54	-0.163	5.22E-05
SNHG12	PRRG4	-0.164	4.63E-05
SNHG12	G2E3	-0.164	4.69E-05
SNHG12	UFL1	-0.164	4.66E-05
SNHG12	UCHL3	-0.164	4.66E-05
SNHG12	STK38L	-0.165	4.30E-05
SNHG12	ADD1	-0.165	4.14E-05
SNHG12	TRIM33	-0.165	3.97E-05
SNHG12	ID4	-0.165	3.96E-05
SNHG12	ARID5B	-0.166	3.71E-05
SNHG12	MECP2	-0.166	3.70E-05
SNHG12	RAP1A	-0.166	3.57E-05
SNHG12	YY1	-0.167	3.37E-05

SNHG12	SETX	-0.168	3.06E-05
SNHG12	MIER3	-0.168	2.89E-05
SNHG12	HIPK2	-0.169	2.71E-05
SNHG12	DIS3	-0.169	2.59E-05
SNHG12	ADIPOR2	-0.169	2.62E-05
SNHG12	YIPF4	-0.169	2.60E-05
SNHG12	VGLL3	-0.17	2.40E-05
SNHG12	ZFYVE16	-0.17	2.44E-05
SNHG12	DNAJA3	-0.171	2.25E-05
SNHG12	UBE4A	-0.171	2.20E-05
SNHG12	AMMECR1	-0.172	1.97E-05
SNHG12	DNAJC13	-0.172	1.91E-05
SNHG12	TMTC3	-0.173	1.66E-05
SNHG12	MME	-0.173	1.73E-05
SNHG12	BTBD7	-0.174	1.60E-05
SNHG12	ACTR2	-0.174	1.59E-05
SNHG12	LRRFIP1	-0.174	1.50E-05
SNHG12	PHAX	-0.175	1.34E-05
SNHG12	SOCS5	-0.175	1.29E-05
SNHG12	SYNCRIP	-0.176	1.16E-05
SNHG12	DR1	-0.176	1.16E-05
SNHG12	CYP1B1	-0.177	1.09E-05
SNHG12	PALLD	-0.177	1.10E-05
SNHG12	EGR1	-0.178	1.00E-05
SNHG12	ELF1	-0.179	8.78E-06
SNHG12	CREBRF	-0.179	8.21E-06
SNHG12	GABPA	-0.18	7.75E-06
SNHG12	ARL13B	-0.18	7.64E-06
SNHG12	JAK1	-0.181	6.97E-06
SNHG12	CA12	-0.181	6.97E-06
SNHG12	ATP2B1	-0.182	5.92E-06
SNHG12	KAT6A	-0.183	5.60E-06
SNHG12	EEF1A1	-0.186	3.68E-06
SNHG12	SLC26A2	-0.186	3.51E-06
SNHG12	C6orf106	-0.187	3.20E-06
SNHG12	PSD3	-0.187	3.35E-06
SNHG12	FZD3	-0.188	2.78E-06
SNHG12	IGFBP5	-0.188	2.80E-06
SNHG12	SMAD5	-0.189	2.57E-06
SNHG12	SELENOF	-0.19	2.23E-06
SNHG12	TROVE2	-0.191	2.02E-06
SNHG12	HSPA13	-0.191	2.07E-06
SNHG12	EFR3A	-0.191	1.88E-06
SNHG12	ZBTB38	-0.192	1.68E-06
SNHG12	THBS1	-0.192	1.74E-06
SNHG12	HDGFL3	-0.192	1.73E-06
SNHG12	NF1	-0.193	1.64E-06
SNHG12	KDSR	-0.193	1.58E-06
SNHG12	RECK	-0.194	1.36E-06
SNHG12	MTFMT	-0.195	1.19E-06
SNHG12	TAOK1	-0.195	1.15E-06
SNHG12	USP13	-0.196	1.05E-06
SNHG12	AFF4	-0.197	8.97E-07
SNHG12	JADE1	-0.197	9.61E-07
SNHG12	NRIP1	-0.197	9.59E-07
SNHG12	EXOC8	-0.197	8.89E-07
SNHG12	VANGL1	-0.197	9.44E-07
SNHG12	SFMBT1	-0.198	7.79E-07

SNHG12	KAT5	-0.198	7.67E-07
SNHG12	NEK7	-0.2	6.58E-07
SNHG12	KLF9	-0.2	6.16E-07
SNHG12	SOBP	-0.2	5.88E-07
SNHG12	FBXO11	-0.203	4.06E-07
SNHG12	AASDH	-0.203	4.24E-07
SNHG12	KLHL24	-0.204	3.87E-07
SNHG12	CDV3	-0.204	3.66E-07
SNHG12	RAB4A	-0.204	3.57E-07
SNHG12	CDC42EP3	-0.205	3.06E-07
SNHG12	CUX1	-0.205	3.24E-07
SNHG12	THUMPD1	-0.205	3.38E-07
SNHG12	HOXA11	-0.205	3.28E-07
SNHG12	CXXC5	-0.206	2.75E-07
SNHG12	SPART	-0.206	2.97E-07
SNHG12	NFAT5	-0.206	2.74E-07
SNHG12	ZFP1	-0.207	2.53E-07
SNHG12	SNAP29	-0.207	2.56E-07
SNHG12	FZD6	-0.207	2.42E-07
SNHG12	ZMAT2	-0.207	2.38E-07
SNHG12	TCF12	-0.207	2.37E-07
SNHG12	EIF5A2	-0.208	2.28E-07
SNHG12	SMAD4	-0.209	1.91E-07
SNHG12	TRIM24	-0.209	1.76E-07
SNHG12	FUBP3	-0.21	1.65E-07
SNHG12	TOP1	-0.211	1.41E-07
SNHG12	ASH1L	-0.211	1.46E-07
SNHG12	RPS6KA3	-0.211	1.37E-07
SNHG12	ELMOD2	-0.211	1.39E-07
SNHG12	NDUFA8	-0.213	1.14E-07
SNHG12	IGF1R	-0.213	1.09E-07
SNHG12	SMAD2	-0.213	1.05E-07
SNHG12	FOXN3	-0.214	9.76E-08
SNHG12	ARID1B	-0.214	8.74E-08
SNHG12	PDIK1L	-0.214	9.71E-08
SNHG12	HIP1	-0.215	8.05E-08
SNHG12	ACTN4	-0.215	8.53E-08
SNHG12	STX12	-0.215	8.02E-08
SNHG12	YWHAQ	-0.217	5.96E-08
SNHG12	HDAC2	-0.219	4.86E-08
SNHG12	GALNT4	-0.219	4.51E-08
SNHG12	ZNF35	-0.22	3.87E-08
SNHG12	ARL2BP	-0.22	3.89E-08
SNHG12	TANC1	-0.22	4.21E-08
SNHG12	IL6ST	-0.221	3.44E-08
SNHG12	METAP1	-0.221	3.43E-08
SNHG12	SERBP1	-0.222	2.80E-08
SNHG12	UBXN2B	-0.223	2.40E-08
SNHG12	FRS2	-0.223	2.58E-08
SNHG12	LIN7C	-0.223	2.40E-08
SNHG12	RBL2	-0.224	2.03E-08
SNHG12	SCAMP2	-0.224	2.33E-08
SNHG12	PUM2	-0.224	2.10E-08
SNHG12	APC	-0.224	2.15E-08
SNHG12	ADIPOR1	-0.225	1.86E-08
SNHG12	PPP3CA	-0.226	1.57E-08
SNHG12	HNRNPR	-0.226	1.69E-08
SNHG12	CRK	-0.226	1.74E-08

SNHG12	PRRC2B	-0.226	1.66E-08
SNHG12	PGAP1	-0.227	1.31E-08
SNHG12	TMOD3	-0.227	1.37E-08
SNHG12	DDX3X	-0.228	1.18E-08
SNHG12	TOGARAM	-0.228	1.14E-08
SNHG12	MTF1	-0.228	1.28E-08
SNHG12	SP2	-0.229	1.04E-08
SNHG12	PGRMC1	-0.23	8.79E-09
SNHG12	PHF6	-0.23	9.48E-09
SNHG12	PCNX4	-0.231	7.90E-09
SNHG12	SLC25A44	-0.231	7.19E-09
SNHG12	DHTKD1	-0.231	7.90E-09
SNHG12	SMC2	-0.232	6.65E-09
SNHG12	ZXDB	-0.233	5.78E-09
SNHG12	SPOPL	-0.234	5.07E-09
SNHG12	CDC14A	-0.235	4.15E-09
SNHG12	UBE3A	-0.235	3.94E-09
SNHG12	CADM1	-0.235	4.01E-09
SNHG12	TEAD1	-0.235	4.34E-09
SNHG12	SLC25A24	-0.237	3.25E-09
SNHG12	PLPP3	-0.237	2.82E-09
SNHG12	NACC2	-0.237	2.80E-09
SNHG12	HIPK1	-0.237	2.92E-09
SNHG12	PAPOLA	-0.237	3.17E-09
SNHG12	ZNF268	-0.238	2.47E-09
SNHG12	CTNND1	-0.238	2.77E-09
SNHG12	BLOC1S5-	-0.239	2.29E-09
SNHG12	CAMSAP2	-0.239	2.12E-09
SNHG12	NR2F2	-0.24	1.96E-09
SNHG12	SBF2	-0.241	1.61E-09
SNHG12	CAMK2N1	-0.242	1.45E-09
SNHG12	LONP2	-0.242	1.46E-09
SNHG12	SESN3	-0.242	1.40E-09
SNHG12	COL4A3BF	-0.242	1.45E-09
SNHG12	APPBP2	-0.242	1.43E-09
SNHG12	SLBP	-0.243	1.17E-09
SNHG12	YME1L1	-0.243	1.19E-09
SNHG12	NPR3	-0.243	1.24E-09
SNHG12	IQGAP1	-0.243	1.16E-09
SNHG12	C5orf24	-0.243	1.22E-09
SNHG12	TNPO1	-0.244	1.02E-09
SNHG12	CASC4	-0.244	9.85E-10
SNHG12	CCNG1	-0.246	7.50E-10
SNHG12	ERBIN	-0.247	6.27E-10
SNHG12	PAK2	-0.247	6.49E-10
SNHG12	NLN	-0.248	5.11E-10
SNHG12	STEAP2	-0.249	4.70E-10
SNHG12	PTPRJ	-0.25	3.58E-10
SNHG12	ZNF620	-0.25	3.78E-10
SNHG12	SDAD1	-0.25	3.56E-10
SNHG12	CHURC1	-0.25	3.89E-10
SNHG12	FNDC3A	-0.251	2.95E-10
SNHG12	DUSP16	-0.251	3.00E-10
SNHG12	PUM1	-0.251	3.03E-10
SNHG12	SSR1	-0.251	3.02E-10
SNHG12	DNAJC21	-0.251	3.36E-10
SNHG12	ABI2	-0.251	3.31E-10
SNHG12	TMBIM4	-0.252	2.59E-10

SNHG12	BECN1	-0.252	2.85E-10
SNHG12	GOLIM4	-0.253	2.32E-10
SNHG12	OCRL	-0.253	2.39E-10
SNHG12	CERS6	-0.254	1.91E-10
SNHG12	ARMC1	-0.254	2.03E-10
SNHG12	TRAPPC10	-0.255	1.56E-10
SNHG12	MAP1LC3B	-0.256	1.41E-10
SNHG12	AZIN1	-0.256	1.33E-10
SNHG12	PRTG	-0.257	1.13E-10
SNHG12	CREB3L2	-0.257	1.23E-10
SNHG12	ITGA2	-0.257	1.18E-10
SNHG12	CMTM6	-0.259	7.78E-11
SNHG12	KAT2B	-0.26	7.23E-11
SNHG12	GLG1	-0.26	7.32E-11
SNHG12	MBIP	-0.26	6.64E-11
SNHG12	HNRNPK	-0.261	5.49E-11
SNHG12	IBTK	-0.261	6.07E-11
SNHG12	SATB1	-0.261	5.92E-11
SNHG12	TOB2	-0.262	4.98E-11
SNHG12	ZBTB33	-0.262	4.40E-11
SNHG12	CCDC117	-0.263	3.72E-11
SNHG12	KPNA6	-0.264	3.42E-11
SNHG12	LYSMD3	-0.264	3.48E-11
SNHG12	RALGPS2	-0.265	2.84E-11
SNHG12	GNAQ	-0.266	2.29E-11
SNHG12	TMEM167A	-0.267	2.04E-11
SNHG12	KIAA0040	-0.267	2.13E-11
SNHG12	KIF1B	-0.267	1.85E-11
SNHG12	ANKFY1	-0.269	1.30E-11
SNHG12	WWC1	-0.269	1.41E-11
SNHG12	WDFY3	-0.269	1.46E-11
SNHG12	ZMAT3	-0.27	1.16E-11
SNHG12	JKAMP	-0.27	1.08E-11
SNHG12	MTMR9	-0.271	9.84E-12
SNHG12	DDX6	-0.271	1.02E-11
SNHG12	UBR1	-0.271	1.05E-11
SNHG12	SNAPC5	-0.272	7.77E-12
SNHG12	RRM2B	-0.272	7.74E-12
SNHG12	MKLN1	-0.273	6.21E-12
SNHG12	PAIP1	-0.275	4.46E-12
SNHG12	CALM2	-0.275	4.27E-12
SNHG12	ATF1	-0.276	3.63E-12
SNHG12	VPS50	-0.276	3.58E-12
SNHG12	EIF3A	-0.277	3.09E-12
SNHG12	ZNF618	-0.277	3.12E-12
SNHG12	RYBP	-0.279	2.29E-12
SNHG12	RAB6A	-0.279	2.30E-12
SNHG12	METTL9	-0.279	2.08E-12
SNHG12	LYRM7	-0.28	1.94E-12
SNHG12	PRKDC	-0.28	1.98E-12
SNHG12	ZNF106	-0.282	1.27E-12
SNHG12	CLOCK	-0.284	8.04E-13
SNHG12	ACVR2A	-0.284	8.31E-13
SNHG12	LRP12	-0.285	6.54E-13
SNHG12	VPS26A	-0.286	5.86E-13
SNHG12	RHOBTB3	-0.286	6.21E-13
SNHG12	KRAS	-0.287	4.80E-13
SNHG12	SPPL2A	-0.287	4.37E-13

SNHG12	ATRX	-0.287	4.44E-13
SNHG12	RBMXL1	-0.288	3.74E-13
SNHG12	CTNNB1	-0.288	3.78E-13
SNHG12	MAPK1	-0.288	3.87E-13
SNHG12	SLC5A3	-0.289	3.02E-13
SNHG12	DYM	-0.29	2.64E-13
SNHG12	PDPK1	-0.29	2.74E-13
SNHG12	PURA	-0.29	2.75E-13
SNHG12	FAM120A	-0.29	2.83E-13
SNHG12	VPS36	-0.291	2.16E-13
SNHG12	RAB21	-0.291	2.31E-13
SNHG12	SERP1	-0.291	2.15E-13
SNHG12	SHOC2	-0.292	1.70E-13
SNHG12	PDLIM5	-0.292	1.75E-13
SNHG12	CGGBP1	-0.292	1.69E-13
SNHG12	IPO7	-0.293	1.37E-13
SNHG12	ETF1	-0.293	1.38E-13
SNHG12	CDYL	-0.293	1.43E-13
SNHG12	YAP1	-0.293	1.49E-13
SNHG12	GPD2	-0.294	1.16E-13
SNHG12	HUWE1	-0.295	9.07E-14
SNHG12	MTUS1	-0.295	9.13E-14
SNHG12	PLAG1	-0.296	7.78E-14
SNHG12	ARMT1	-0.297	7.20E-14
SNHG12	XIAP	-0.298	5.51E-14
SNHG12	LMBRD2	-0.298	4.97E-14
SNHG12	DENND1B	-0.298	5.34E-14
SNHG12	TMEM50B	-0.298	5.10E-14
SNHG12	TACC1	-0.298	5.81E-14
SNHG12	SMC1A	-0.299	4.75E-14
SNHG12	DAAM1	-0.3	3.39E-14
SNHG12	CISD1	-0.3	3.24E-14
SNHG12	PRKCI	-0.3	3.53E-14
SNHG12	GTF2A1	-0.3	3.39E-14
SNHG12	CDC42	-0.301	2.66E-14
SNHG12	MED13	-0.302	2.53E-14
SNHG12	ZDHHC23	-0.303	1.80E-14
SNHG12	ZFP91	-0.303	1.93E-14
SNHG12	VEZF1	-0.303	1.77E-14
SNHG12	ABHD17B	-0.304	1.44E-14
SNHG12	ARF4	-0.305	1.22E-14
SNHG12	CNIH1	-0.306	9.85E-15
SNHG12	WWC2	-0.307	7.98E-15
SNHG12	ZBTB10	-0.307	8.74E-15
SNHG12	XPO7	-0.307	9.20E-15
SNHG12	PCNX1	-0.308	6.38E-15
SNHG12	EXOC5	-0.308	6.83E-15
SNHG12	CNN3	-0.309	6.09E-15
SNHG12	CSDE1	-0.31	4.81E-15
SNHG12	POLR2M	-0.31	4.13E-15
SNHG12	HSPH1	-0.31	4.87E-15
SNHG12	SYAP1	-0.311	3.99E-15
SNHG12	ABRAXAS2	-0.311	3.35E-15
SNHG12	USP10	-0.312	2.73E-15
SNHG12	GSK3B	-0.313	2.20E-15
SNHG12	MAL2	-0.313	2.13E-15
SNHG12	FBXL4	-0.314	1.81E-15
SNHG12	CTCF	-0.314	1.91E-15

SNHG12	VGLL4	-0.316	1.21E-15
SNHG12	ADRB1	-0.316	1.30E-15
SNHG12	ASXL2	-0.317	1.02E-15
SNHG12	PIK3C2A	-0.318	8.67E-16
SNHG12	MYO1D	-0.319	6.38E-16
SNHG12	UEVLD	-0.32	5.41E-16
SNHG12	NUS1	-0.32	4.68E-16
SNHG12	IMPAD1	-0.32	4.68E-16
SNHG12	KIDINS220	-0.321	3.88E-16
SNHG12	WDR7	-0.323	2.39E-16
SNHG12	IPPK	-0.323	2.77E-16
SNHG12	CRIM1	-0.324	2.08E-16
SNHG12	RAB3IP	-0.324	1.99E-16
SNHG12	MAN1A2	-0.325	1.71E-16
SNHG12	ZBTB4	-0.325	1.70E-16
SNHG12	PTP4A1	-0.325	1.65E-16
SNHG12	GAS1	-0.326	1.21E-16
SNHG12	RAB11FIP2	-0.326	1.49E-16
SNHG12	SPTSSA	-0.327	1.11E-16
SNHG12	CALM1	-0.327	1.10E-16
SNHG12	ADD3	-0.327	1.01E-16
SNHG12	CD46	-0.328	8.73E-17
SNHG12	PTPMT1	-0.328	8.91E-17
SNHG12	TSNAX	-0.328	8.88E-17
SNHG12	VTI1A	-0.328	8.45E-17
SNHG12	RNF13	-0.329	6.68E-17
SNHG12	GLCE	-0.331	4.09E-17
SNHG12	NIPSNAP2	-0.331	4.68E-17
SNHG12	VCP	-0.331	4.43E-17
SNHG12	RBM12	-0.331	4.85E-17
SNHG12	PLS1	-0.333	2.73E-17
SNHG12	PARVA	-0.333	2.70E-17
SNHG12	LRRC58	-0.334	2.41E-17
SNHG12	ABHD13	-0.334	2.01E-17
SNHG12	BMPR2	-0.334	2.23E-17
SNHG12	PTCH1	-0.334	2.10E-17
SNHG12	ATP7A	-0.335	1.69E-17
SNHG12	PTPN11	-0.335	1.66E-17
SNHG12	SINHCAF	-0.337	1.14E-17
SNHG12	BCL7A	-0.337	1.09E-17
SNHG12	MAPRE1	-0.338	9.37E-18
SNHG12	TMEM19	-0.339	7.45E-18
SNHG12	ITGA6	-0.34	5.22E-18
SNHG12	THUMPD3	-0.34	4.93E-18
SNHG12	RLIM	-0.34	5.87E-18
SNHG12	ERC1	-0.341	4.14E-18
SNHG12	ZHX3	-0.342	3.57E-18
SNHG12	FAM210B	-0.342	3.25E-18
SNHG12	RPRD1A	-0.342	3.07E-18
SNHG12	H2AFV	-0.342	3.18E-18
SNHG12	PPP2CB	-0.343	2.57E-18
SNHG12	NDUFA10	-0.343	2.70E-18
SNHG12	IER3IP1	-0.344	2.20E-18
SNHG12	ZNF770	-0.344	2.13E-18
SNHG12	FBXO33	-0.344	2.12E-18
SNHG12	USP9X	-0.345	1.43E-18
SNHG12	BCL9	-0.345	1.81E-18
SNHG12	CDC37L1	-0.345	1.67E-18

SNHG12	RAB7A	-0.345	1.48E-18
SNHG12	CUL5	-0.346	1.31E-18
SNHG12	PPP2CA	-0.347	9.44E-19
SNHG12	NR1D2	-0.348	7.66E-19
SNHG12	TOB1	-0.349	5.85E-19
SNHG12	CLINT1	-0.35	4.85E-19
SNHG12	RHOA	-0.35	4.45E-19
SNHG12	ZDHHC5	-0.35	4.91E-19
SNHG12	LONRF2	-0.35	4.87E-19
SNHG12	TRAPPC8	-0.351	3.87E-19
SNHG12	RAB10	-0.351	3.49E-19
SNHG12	UBE2K	-0.352	2.98E-19
SNHG12	C11orf54	-0.354	1.98E-19
SNHG12	BTBD1	-0.354	1.97E-19
SNHG12	MINDY2	-0.355	1.24E-19
SNHG12	UBR3	-0.355	1.29E-19
SNHG12	HOXB9	-0.355	1.26E-19
SNHG12	RUNDC1	-0.357	7.95E-20
SNHG12	CPT1A	-0.358	6.57E-20
SNHG12	LIFR	-0.359	4.49E-20
SNHG12	RNF38	-0.361	2.99E-20
SNHG12	KIF5B	-0.362	2.25E-20
SNHG12	ZFR	-0.363	1.62E-20
SNHG12	TMEM38B	-0.363	1.84E-20
SNHG12	CD2AP	-0.363	1.95E-20
SNHG12	CSTF2T	-0.364	1.25E-20
SNHG12	C5orf22	-0.364	1.30E-20
SNHG12	ZNRF2	-0.365	1.19E-20
SNHG12	C6orf120	-0.365	1.21E-20
SNHG12	PTPN13	-0.365	9.66E-21
SNHG12	IRF2BPL	-0.366	8.71E-21
SNHG12	ARFGEF2	-0.366	7.35E-21
SNHG12	NFE2L2	-0.367	6.51E-21
SNHG12	CAPZA2	-0.367	6.80E-21
SNHG12	EID1	-0.367	5.87E-21
SNHG12	MAPKAP1	-0.368	5.04E-21
SNHG12	WNK1	-0.368	5.25E-21
SNHG12	NTN4	-0.368	5.17E-21
SNHG12	DNAJA2	-0.369	4.21E-21
SNHG12	DNAJC6	-0.37	2.73E-21
SNHG12	MARVELD:	-0.37	3.26E-21
SNHG12	SOS2	-0.373	1.47E-21
SNHG12	LYPLA1	-0.375	7.22E-22
SNHG12	FBXW11	-0.375	7.26E-22
SNHG12	ZNRF3	-0.379	2.86E-22
SNHG12	ARL5A	-0.38	1.86E-22
SNHG12	MIB1	-0.383	1.01E-22
SNHG12	THAP10	-0.383	8.65E-23
SNHG12	MRPL44	-0.384	6.71E-23
SNHG12	RAB5A	-0.384	7.53E-23
SNHG12	OSBPL8	-0.385	5.65E-23
SNHG12	CHMP2B	-0.385	4.97E-23
SNHG12	PRKAA2	-0.385	5.67E-23
SNHG12	CHM	-0.388	2.36E-23
SNHG12	GSKIP	-0.39	1.18E-23
SNHG12	STAM2	-0.391	9.27E-24
SNHG12	STAU2	-0.392	7.00E-24
SNHG12	WDR48	-0.393	5.44E-24

SNHG12	CYP2U1	-0.394	3.62E-24
SNHG12	SLAIN2	-0.394	3.86E-24
SNHG12	CPEB3	-0.394	3.55E-24
SNHG12	HOXA7	-0.395	3.45E-24
SNHG12	PTPN4	-0.395	2.83E-24
SNHG12	TRIP11	-0.396	2.04E-24
SNHG12	LAPTM4B	-0.397	1.94E-24
SNHG12	RRAGD	-0.397	1.87E-24
SNHG12	PAPPA	-0.397	1.52E-24
SNHG12	RAD23B	-0.397	1.67E-24
SNHG12	SEMA6D	-0.397	1.46E-24
SNHG12	SOWAHC	-0.399	1.01E-24
SNHG12	PTBP3	-0.4	6.99E-25
SNHG12	DDX1	-0.401	4.93E-25
SNHG12	LMBR1	-0.402	4.50E-25
SNHG12	USF3	-0.402	4.37E-25
SNHG12	ATP1B1	-0.404	2.38E-25
SNHG12	NCOA1	-0.409	5.20E-26
SNHG12	LRP10	-0.409	4.67E-26
SNHG12	TM9SF3	-0.41	3.94E-26
SNHG12	ACSL1	-0.41	3.11E-26
SNHG12	OGFOD1	-0.411	2.28E-26
SNHG12	ZMYND11	-0.411	2.37E-26
SNHG12	SNX4	-0.411	2.98E-26
SNHG12	LANCL1	-0.411	3.04E-26
SNHG12	ACBD5	-0.414	1.07E-26
SNHG12	NCOA7	-0.414	1.12E-26
SNHG12	RANBP2	-0.414	1.00E-26
SNHG12	UHMK1	-0.415	7.49E-27
SNHG12	TBC1D9	-0.417	4.63E-27
SNHG12	RMND5A	-0.42	1.52E-27
SNHG12	GPHN	-0.42	1.49E-27
SNHG12	ATAD1	-0.422	8.47E-28
SNHG12	CD164	-0.427	1.58E-28
SNHG12	CPEB4	-0.428	1.42E-28
SNHG12	GLYR1	-0.428	1.27E-28
SNHG12	FBXO21	-0.429	8.89E-29
SNHG12	FAM171A1	-0.43	6.15E-29
SNHG12	GDE1	-0.43	6.10E-29
SNHG12	UTP14C	-0.433	2.55E-29
SNHG12	NCOA4	-0.434	1.88E-29
SNHG12	SEPHS1	-0.435	1.19E-29
SNHG12	CLCN5	-0.436	1.10E-29
SNHG12	SECISBP2L	-0.436	8.84E-30
SNHG12	KLHL15	-0.437	8.01E-30
SNHG12	ACSL4	-0.438	5.35E-30
SNHG12	ASB8	-0.438	5.29E-30
SNHG12	SACM1L	-0.438	4.50E-30
SNHG12	MRFAP1	-0.439	3.62E-30
SNHG12	SNX18	-0.44	2.57E-30
SNHG12	ARHGAP5	-0.44	2.56E-30
SNHG12	SLC25A25	-0.441	1.81E-30
SNHG12	KIAA0232	-0.444	7.35E-31
SNHG12	CCDC186	-0.445	4.47E-31
SNHG12	AUH	-0.447	2.41E-31
SNHG12	MECOM	-0.448	1.84E-31
SNHG12	DYNLT3	-0.448	1.80E-31
SNHG12	NT5DC1	-0.449	1.36E-31

SNHG12	RAB14	-0.449	1.25E-31
SNHG12	ERLIN2	-0.449	1.43E-31
SNHG12	CROT	-0.45	8.36E-32
SNHG12	ZHX1	-0.451	6.85E-32
SNHG12	ANKRD46	-0.452	4.58E-32
SNHG12	AKIRIN1	-0.452	4.44E-32
SNHG12	BBX	-0.453	2.75E-32
SNHG12	SLC44A1	-0.454	1.99E-32
SNHG12	FAM169A	-0.454	1.85E-32
SNHG12	YPEL5	-0.455	1.41E-32
SNHG12	NAA50	-0.456	9.24E-33
SNHG12	ABHD2	-0.457	7.52E-33
SNHG12	USP38	-0.459	3.71E-33
SNHG12	PBX1	-0.461	1.82E-33
SNHG12	PRKAR2A	-0.462	1.33E-33
SNHG12	RTN4	-0.462	1.27E-33
SNHG12	ALDH5A1	-0.463	9.07E-34
SNHG12	NIPAL1	-0.465	4.74E-34
SNHG12	SLC48A1	-0.465	3.99E-34
SNHG12	MFAP3L	-0.466	3.35E-34
SNHG12	RBBP5	-0.474	1.30E-35
SNHG12	WASL	-0.478	2.97E-36
SNHG12	IVD	-0.479	2.11E-36
SNHG12	PRMT6	-0.486	1.41E-37
SNHG12	HACD2	-0.486	1.34E-37
SNHG12	CAB39	-0.486	1.54E-37
SNHG12	KLHL8	-0.488	6.27E-38
SNHG12	TMBIM6	-0.489	4.72E-38
SNHG12	SLC31A1	-0.492	1.61E-38
SNHG12	ICMT	-0.492	1.37E-38
SNHG12	TMEM9B	-0.494	6.91E-39
SNHG12	TMEM184	-0.495	3.63E-39
SNHG12	SCP2	-0.495	5.00E-39
SNHG12	VTI1B	-0.5	5.09E-40
SNHG12	ALDH9A1	-0.504	1.30E-40
SNHG12	ABHD17C	-0.505	8.35E-41
SNHG12	AK3	-0.505	6.16E-41
SNHG12	SEC62	-0.506	4.58E-41
SNHG12	RDH11	-0.507	2.82E-41
SNHG12	CLTC	-0.51	9.13E-42
SNHG12	YWHAB	-0.515	1.11E-42
SNHG12	CTDSPL	-0.516	8.55E-43
SNHG12	ISOC1	-0.516	7.87E-43
SNHG12	HSD17B12	-0.524	1.99E-44
SNHG12	HSDL2	-0.525	1.28E-44
SNHG12	RNF11	-0.529	2.74E-45
SNHG12	GLRX5	-0.529	1.92E-45
SNHG12	SC5D	-0.529	2.77E-45
SNHG12	DLAT	-0.534	2.59E-46
SNHG12	ACSL3	-0.535	1.92E-46
SNHG12	CDS1	-0.538	3.47E-47
SNHG12	DBT	-0.54	1.86E-47
SNHG12	MPP5	-0.541	1.08E-47
SNHG12	NR3C2	-0.547	4.49E-49
SNHG12	PCYOX1	-0.548	3.62E-49
SNHG12	NNT	-0.558	2.68E-51
SNHG12	MUT	-0.562	3.44E-52
SNHG12	DLD	-0.586	1.34E-57

SNHG12	ATP6V1A	-0.595	1.03E-59
SNHG12	HACD3	-0.614	1.41E-64
