# Supplementary material for: Long Non-coding RNA SNHG12 Functions as a Competing Endogenous RNA to Regulate MDM4 Expression by Sponging miR-129-5p in Clear Cell Renal Cell Carcinoma
Source: Front Oncol. 2019 Nov 22;9:1260. doi: 10.3389/fonc.2019.01260 (PMC6882951; doi:10.3389/fonc.2019.01260)
Supplement: Supplementary file 2 [file Image_1.pdf]

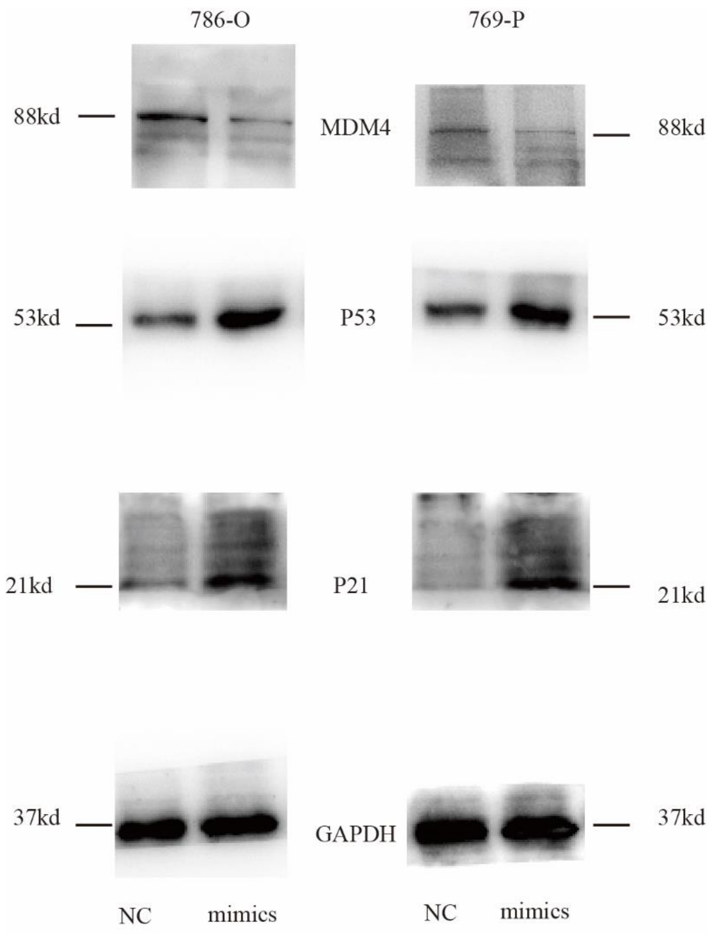

Figure S1 Original image files for the blots in Figure 6F.

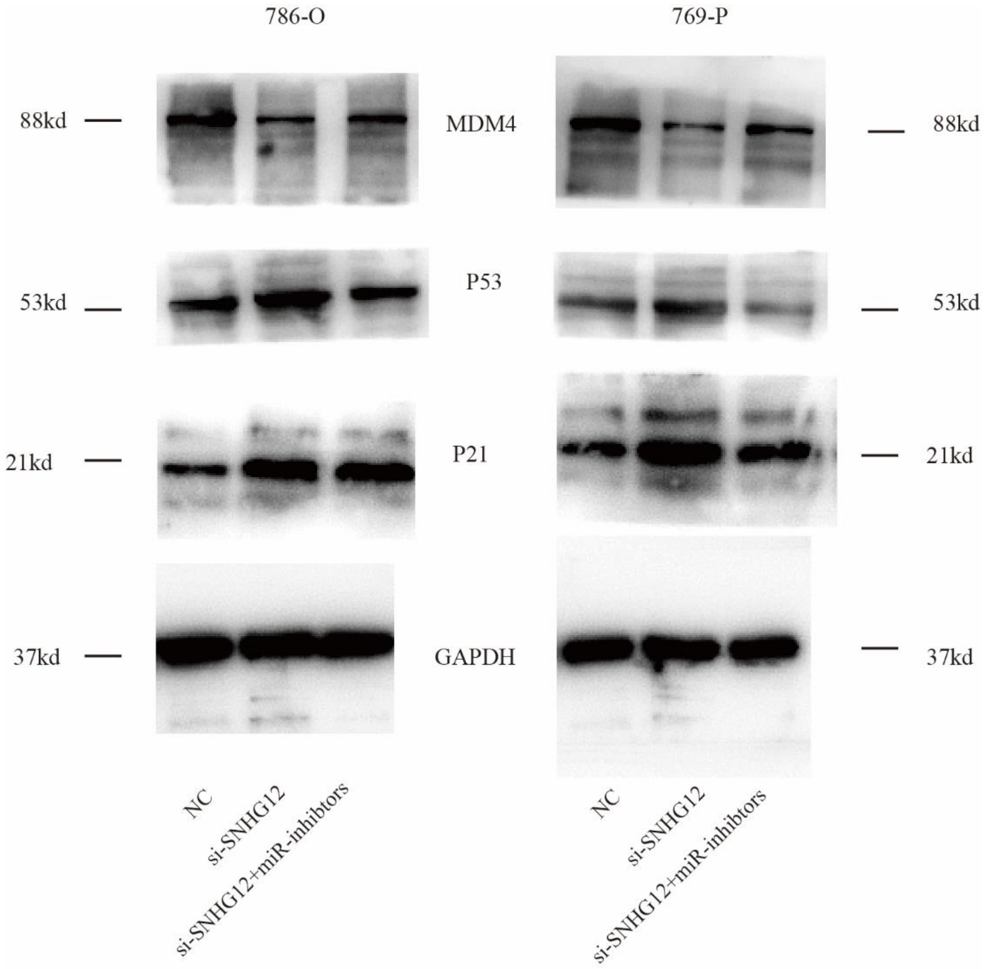

Figure S2 Original image files for the blots in Figure 6H.
